# Xanthones and Quinolones Derivatives Produced by the Deep-Sea-Derived Fungus *Penicillium* sp. SCSIO Ind16F01

**DOI:** 10.3390/molecules22121999

**Published:** 2017-12-07

**Authors:** Feng-an Liu, Xiuping Lin, Xuefeng Zhou, Minghao Chen, Xiuling Huang, Bin Yang, Huaming Tao

**Affiliations:** 1 School of Traditional Chinese Medicine, Southern Medical University, Guangzhou 510515 China; liufeng.an@outlook.com (F.-a.L.); chen.minghao@haotmail.com (M.C.), aguohxl@sina.com (X.H.); 2 CAS Key Laboratory of Tropical Marine Bio-resources and Ecology/Guangdong Key Laboratory of Marine Materia Medica, South China Sea Institute of Oceanology, Chinese Academy of Sciences, Guangzhou 510301 China; xiupinglin@hotmail.com (X.L.); xfzhou@scsio.ac.cn (X.Z.)

**Keywords:** marine-derived fungus, *Penicillium* sp. SCSIO Ind16F01, xanthone, chromone, *N*-methyl quinolone lactams

## Abstract

Chemical investigation of the fungus *Penicillium* sp. SCSIO Ind16F01 derived from deep-sea sediment sample afforded a new xanthone, 3,8-dihydroxy-2-methyl-9-oxoxanthene-4-carboxylic acid methyl ester (**1**) and a new chromone, coniochaetone J (**2**), together with three known xanthones, 8-hydroxy-6-methyl-9-oxo-9*H*-xanthene-1-carboxylic acid methyl ester (**3**), 7,8-dihydroxy-6-methyl-9-oxo-9*H*-xanthene-1-carboxylic acid methyl ester (**4**), 1,6,8-trihydroxy-3-(hydroxymethyl)anthraquinone (**5**), three known chromones, coniochaetone B (**6**), citrinolactones B (**7**), epiremisporine B (**8**), and four reported rare class of *N*-methyl quinolone lactams: quinolactacins B (**9**), C1 (**10**), and C2 (**11**), and quinolonimide (**12**). The structures of new compounds were determined by analysis of the NMR and MS spectroscopic data. Those isolated compounds were evaluated for their antiviral (EV71 and H3N2) and cytotoxic activities.

## 1. Introduction

Marine microorganisms, especially the marine fungi, tend to produce structurally unique and biologically active natural products which have been documented in recent years [1,2,3,4]. Recently, microorganisms from deep-sea habitats, including the hydrothermal vents, have become an interesting and newly emerging source of novel bioactive compounds, which play an important role in drug discovery [5].

**Figure 1:**
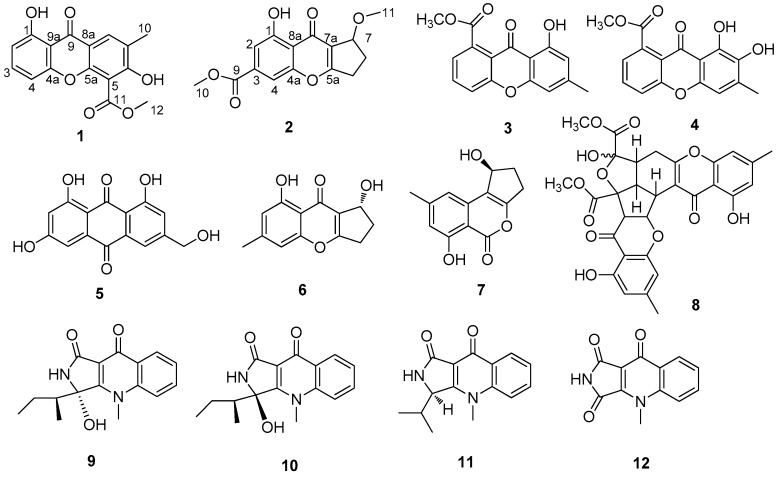
Structures of metabolites **1**–**12**.

As a part of our progressive program to explore the antiviral potential of marine fungi, the secondary metabolites of the strain SCSIO Ind16F01 were examined. Two new compounds: 3,8-dihydroxy-2-methyl-9-oxoxanthene-4-carboxylic acid methyl ester (**1**) and a new chromone, coniochaetone J (**2**), together with ten known compounds: 8-hydroxy-6-methyl-9-oxo-9*H*-xanthene-1-carboxylic acid methyl ester (**3**), 7,8-dihydroxy-6-methyl-9-oxo-9*H*-xanthene-1-carboxylic acid methyl ester (**4**), 1,6,8-trihydroxy-3-(hydroxymethyl)anthraquinone (**5**), coniochaetone B (**6**), citrinolactones B (**7**), epiremisporine B (**8**), quinolactacins C1 (**9**), C2 (**10**), and B (**11**), and quinolonimide (**12**) (Figure 1) were isolated from the ethyl acetate crude extracts of the rice medium. Their structures were established on the basis of extensive spectroscopic techniques. The isolated compounds were evaluated for their anti-H3N2, anti-enterovirus 71 (EV71) and cytotoxic activities, respectively. We present herein the fermentation, isolation, structural elucidation, bioactive assay of compounds **1**–**12**.

## 2. Results

**Figure 2:**
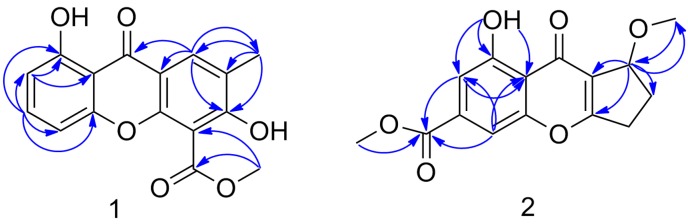
Key HMBC (half arrows) correlations of compounds **1** and **2**.

Compound **1** was isolated as white amorphous solid. Based on the HRESIMS ion peak at *m*/*z* 299.0569 [M − H]^−^ (calcd. for 299.0561), the molecular formula was established as C_16_H_12_O_6_ indicating eleven degrees of unsaturations (Figure S5). The ^1^H NMR spectrum of **1** showed characteristic signals for three vicinal protons as an AMX spin system at *δ*_H_ 6.80 (1H, dd, *J* = 0.7, 8.5 H_Z_, H-4), 7.05 (1H, dd, *J* = 0.7, 8.5 H_Z_, H-2), 7.72 (1H, t, *J* = 8.5 H_Z_, H-3), and one olefinic proton at *δ*_H_ 7.57 (1H, d, *J* = 1.0 H_Z_, H-8) (Figure S1). Additionally, one intramolecularly hydrogen-bonded hydroxyl group at *δ*_H_ 12.32, a methoxy singlet at *δ*_H_ 3.87 and an aromatic methyl singlet at *δ*_H_ 2.38 were observed. The ^13^C NMR data of **1**, with the aid of DEPT and HSQC experiments (Table 1, Figures S2 and S3), showed resonances for two methyl groups (one methoxy), twelve aromatic carbons with four protonated, one carboxylic carbon (*δ*_C_ 167.0), and one α,β-unsaturated ketone carbon (*δ*_C_ 180.5). These data showed great similarities to those of 2,8-dihydroxy-3-methyl-9-oxoxanthene-1-carboxylic acid methyl ester [6] with the difference in ring C. The HMBC correlations of H-8 to C-6, C-5a, C-8a, and C-9, CH_3_-12 to C-11 and C-5, CH_3_-10 to C-6, C-7 and C-8 revealed that a phenolic OH was attached to C-6 and a carboxymethoxy moiety was attached to C-5 (Figure 2 and Figure S4). The positioning of the phenolic OH at C-6 was further supported by the base peak observed at *m*/*z* 268 [M^+^ − MeOH] (Figure S8), as previously illustrated for 2,8-dihydroxy-3-methyl-9-oxoxanthene-1-carboxylic acid methyl ester [6]. Thus, compound **1** was suggested to be 1,6-dihydroxy-7-methyl-9-oxoxanthene-5-carboxylic acid methyl ester.

Compound **2** was isolated as white amorphous solid. Its molecular formula was assigned as C_28_H_36_N_3_O_5_ based on the HRESIMS at *m/z*: 313.0687 [M + Na]^+^ (calcd. for C_15_H_14_NaO_6_ 313.0683), accounting for nine degrees of unsaturation (Figure S13). The ^1^H NMR spectrum of **2** showed two olefinic proton at *δ*_H_ 7.41 (1H, s, H-2) and 7.54 (1H, s, H-4) one oxygenated methine at *δ*_H_ 4.94 (1H, d, *J* = 6.5 Hz, H-7), and two oxygenated methyl at *δ*_H_ 3.94 (3H, s, H-11) and 3.49 (3H, s, H-10) (Figure S9). Its ^13^C NMR (DEPT) spectrum include signals for two methyl groups, two methines, one oxygenated methylene, eight aromatic/olefinic carbons with two protonated, one carboxylic carbon (*δ*_C_ 165.4), and one α, β-unsaturated ketone carbon (*δ*_C_ 181.0). The ^1^H and ^13^C NMR spectra of **2** are similar to those of coniochaetone B, with the exception that the methyl at C-3 of the latter were replaced by a carboxymethoxy moiety and the hydroxy at C-7 was replaced by a methoxy group (Table 1, Figures S10 and S11). The HMBC correlations of OH-1 to C-1, C-2, and C-8a, H-2 to C-3, C-1, C-8a, and C-9, H-4 to C-4a, C-2, C-8a and C-9, CH_3_-10 to C-9, and CH_3_-11 to C-7 revealed the positioning of the phenolic OH at C-1, a carboxymethoxy moiety at C-3 and a methoxy at C-7 (Figure S12). The configuration of similars at C-7 were determined on the basis of optical rotation in the literature, which the 7*S* absolute configuration showed negative optical rotation and 7*R* absolute configuration showed positive sign [7,8]. However, compound **2** didn’t show optical rotation value or cotton effect in the CD spectrum. Thus, compound **2** was a racemate.

By comparing the ^1^H, ^13^C NMR and MS data with the literature values, the known compounds were identified as 8-hydroxy-6-methyl-9-oxo-9*H*-xanthene-1-carboxylic acid methyl ester (**3**) [9], 7,8-dihydroxy-6-methyl-9-oxo-9*H*-xanthene-1-carboxylic acid methyl ester (**4**) [10], 1,6,8-trihydroxy-3-(hydroxymethyl)anthraquinone (**5**) [10], coniochaetone B (**6**) [8,10], citrinolactone B (**7**) [11], epiremisporine B (**8**) [7,12], quinolactacins C1 (**9**) [13,14,15], C2 (**10**) [13,14,15], and B (**11**) [16], and quinolonimide (**12**) [15]. The stereochemistry of **6**, **7**, **9** and **11** was confirmed by the optical rotation, and comparison with literature values. Compound **10** was obtained as a mixture of quinolactacins C1 and C2. The configurations of the compound **8** were not determined.

The isolated compounds were evaluated for their antiviral (H3N2 and EV71) and cytotoxic activities (Table S1). Among them, compounds **2** and **8** exhibited weak inhibitory activity against EV71 in vitro, with IC_50_ values of 81.6 and 19.8 μM. In addition, compound **8** also exhibited inhibitory activity against H3N2 with IC_50_ values of 24.1 μM, and cytotoxic effects on the tested cancer cell lines (K562, MCF-7, and SGC7901) with IC_50_ values of 16.6, 16.3, and 15.8 μM, respectively.

## 3. Materials and Methods

**General experimental procedures.** ^1^H-, ^13^C-NMR, DEPT and 2D-NMR spectra were recorded on a Bruker AC 500 NMR spectrometer with TMS as an internal standard. HR-ESI-MS data were measured on a Bruker microTOF-QII mass spectrometer. CD spectra were measured with a Chirascan circular dichroism spectrometer (Applied Photophysics, Surrey, UK). Optical rotation values were measured with a PerkineElmer 341 polarimeter. Column chromatography were performed on silica gel (200–300 mesh; Qingdao Marine Chemical Factory, Qingdao, China), YMC gel (ODS-A, 12 nm, S-50 µm) and Sephadex LH-20 (Amersham Biosciences, Uppsala, Sweden), respectively. The silica gel GF_254_ used for TLC were supplied by the Qingdao Marine Chemical Factory, Qingdao, China. All solvents used were of analytical grade (Tianjin Fuyu Chemical and Industry Factory, Tianjin, China). HPLC was carried on Hitachi L-2400 with YMC ODS column. Spots were detected on TLC under UV light or by heating after spraying with 5% H_2_SO_4_ in EtOH (*v*/*v*).

**Fungal Material.** The culture of *Penicillium* sp. SCSIO Ind16F01 was isolated from a deep-sea sediment sample collected in the Indian Ocean (10.00371667° N, 88.72803333° E) in 2013. The strain (accession No. MF945609) was identified as *Penicillium* sp. based on sequence analysis of the internal transcribed spacer (ITS) region. The DNA was amplified and ITS region sequence was compared with the GenBank database and shared a similarity of 100% with *Penicillium citrinum*. This strain was stored on MB agar slants at 4 °C and then deposited at CAS Key Laboratory of Tropical Marine Bio-resources and Ecology.

**Fermentation and Extraction.** Strain stored on PDA slants at 4 °C was cultured MB agar (malt extract 15 g, sea salt 10 g, agar 15 g, distilled water 1 l, pH 7.4–7.8) plates. Seed medium (malt extract 15 g, sea salt 10 g, distilled water 1 L, pH 7.4–7.8) in 50-mL Erlenmeyer flasks was inoculated with strain SCSIO Ind16F01 and incubated at 25 °C for 48 h on a rotating shaker (180 rpm). Production medium of solid rice in 1000 mL flasks (rice 200 g, NaCl 0.5 g, distilled water 200 mL) was inoculated with 10 mL seed solution. Flasks were incubated at 25 °C under static stations and daylight. After 60 days, cultures from 30 flasks were harvested for the isolation of substances.

The total rice solid culture was crushed and extracted with acetone three times. The acetone extract was evaporated under reduced pressure to afford an aqueous solution, and then the aqueous solution was extracted with EtOAc to yield 62 g of a crude gum. The H_2_O layer (120 g) was further partitioned n-Butyl alcohol to yield n-Butyl alcohol (55.6 g) fractions.

**Isolation and Purification.** The EtOAc portion was subsequently separated by Si gel column chromatography using CHCl_3_–MeOH gradient elution to give forty-one fractions (Fr.1–41). Fr.25 was separated by CC over Sephadex LH-20 (CHCl_3_–MeOH 1:1) to get five subfractions (Fr.25.1–Fr.25.5). Subfraction Fr.25.1 was purified by CC on silica gel eluting with petroleum ether (PE)–ethyl acetate (EtOAc) (4:1) to get **3** (3.3 mg). Fr.33 was separated by CC over Sephadex LH-20 (CHCl_3_–MeOH 1:1) to get six subfractions (Fr.33.1–Fr.33.6). Subfraction Fr.33.4 was purified by CC on silica gel eluting with CHCl_3_–MeOH (30:1) to get **2** (4.3 mg) and **4** (5.4 mg). Fr.37 was separated by CC over Sephadex LH-20 (CHCl_3_–MeOH 1:1) to get five subfractions (Fr.37.1–Fr.37.5). Subfraction Fr.37.5 was purified by CC on silica gel eluting with CHCl_3_–acetone (150:1) to get **5** (4.1 mg) and **7** (9.4 mg). The total *n*-Butyl alcohol extract was subjected to column chromatography (CC) on ODS, eluting with a gradient MeOH–H_2_O (5:95–100:0) to give twenty fractions (Fr.s1–Fr.s20). Fr.s2 was separated by CC over Sephadex LH-20 (CHCl_3_–MeOH 1:1) to get seven subfractions (Fr.s2.1–Fr.s2.7). Fr.s2.1 was purified by semipreparative RP HPLC (58% MeOH in H_2_O) at a flow rate of 3 mL/min to afford **6** (3.5 mg). Fr.s14 was separated by CC over Sephadex LH-20 (CHCl_3_–MeOH 1:1) to get nine subfractions (Fr.s14.1–Fr.s14.9). Fr.s14.1 was purified by semipreparative RP HPLC (50% MeOH in H_2_O) at a flow rate of 3 mL/min to afford **1** (2.8 mg). Fr.s14.3 was purified by semipreparative RP HPLC (60% MeOH in H_2_O) at a flow rate of 3 mL/min to afford **8** (6.6 mg). Fr.s14.4 was purified by semipreparative RP HPLC (30% MeOH in H_2_O) at a flow rate of 3 mL/min to afford **9** (7.8 mg), and **10** (6.1 mg). Fr.s14.6 was purified by semipreparative RP HPLC (50% MeOH in H_2_O) at a flow rate of 3 mL/min to afford **12** (3.8 mg). Fr.s16 was separated by CC over Sephadex LH-20 (CHCl_3_–MeOH 1:1) to get five subfractions (Fr.s16.1–Fr.s16.5). Fr.s16.3 was purified by semipreparative RP HPLC (40% MeOH in H_2_O) at a flow rate of 3 mL/min to afford **11** (4.8 mg).

*3,8-Dihydroxy-2-methyl-9-oxoxanthene-4-carboxylic acid methyl ester* (**1**): White amorphous solid; UV (MeOH) λ_max_ (log ε) 236 (0.52), 261 (0.59), 290 (0.23), 382 (0.16) nm; IR (KBr) ν_max_ 3177, 1690, 1647, 1598, 1468, 1372, 1303, 1241, 1056, 1020, 774 cm^−^1; ^1^H and ^13^C NMR data: see Table 1; HRESIMS: *m/z*: 299.0559 [M − H]^−^ (calcd. for C_16_H_11_O_6_ 299.0561).

*Coniochaetone J* (**2**): White amorphous solid; [*α*${]}_{D}^{25}$ 0 (MeOH; *c* 0.2); UV (MeOH) λ_max_ (log ε) 225 (1.56), 245 (1.78), 347 (0.45) nm; IR (KBr) ν_max_ 3094, 2949, 1721, 1647, 1618, 1448, 1246, 1080, 765 cm^−1^; ^1^H and ^13^C NMR data: see Table 1; HRESIMS: *m/z*: 313.0687 [M + Na]^+^ (calcd. for C_15_H_14_NaO_6_ 313.0683), 603.1473 [2M + Na]^+^ (calcd. for C_30_H_28_NaO_12_ 603.1473).

**Biological Activity.** Cytotoxic assay: All isolated compounds were evaluated against the three human tumor cell lines (K562, MCF-7, SGC7901) according to Bergeron et al. [17]. Cell lines, K562, MCF-7, SGC7901 were purchased from Shanghai Cell Bank, Chinese Academy of Sciences. Cells were routinely grown and maintained in mediums RPMI or DMEM with 10% FBS and with 1% penicillin/streptomycin. All cell lines were incubated in a Thermo/Forma Scientific CO_2_ Water Jacketed Incubator with 5% CO_2_ in air at 37 °C. Cell viability assay was determined by the CCK8 (DOjinDo, Kumamoto, Japan) assay. Cells were seeded at a density of 400–800 cells/ well in 384 well plates and treated with various concentration of compounds or solvent control. After 72 h incubation, CCk8 reagent was added, and absorbance was measured at 450 nm using Envision 2104 multi-label Reader (Perkin Elmer, Waltham, MA, USA). Dose response curves were plotted to determine the IC_50_ values using Prism 5.0 (GraphPad Software Inc., La Jolla, CA, USA).

Antiviral assay: The antiviral activities against H3N2 were evaluated by the CPE inhibition assay in duplicate assay [18]. Oseltamivir was used as the positive control with IC_50_ values of 16.9 nM, respectively. Confluent MDCK cell monolayers were incubated with influenza virus at 37 °C for 1 h. After removing the virus, cells were maintained in infecting media (RPMI 1640, 4 μg/mL of trypsin) containing different concentrations of test compounds. After 48 h of incubation at 37 °C, the cells were fixed with 100 μL of 4% formaldehyde for 20 min at room temperature. After removal of the formaldehyde, the cells were stained with 0.1% crystal violet for 30 min. The plates were washed and dried, and the intensity of crystal violet staining for each well was measured in a microplate reader (Bio-Rad, Hercules, CA, USA) at 570 nm. The IC_50_ was calculated as the compound concentration required to inhibit influenza virus yield at 48 h postinfection by 50%. Tamiflu was used as the positive control, with IC_50_ values of 16.9 and 18.5 nM, respectively.

Anti-EV71 was assayed on Vero cells with the CCK8 (DOjinDo, Kumamoto, Japan) method [19]. Ribavirinwas used as the positive control with an IC_50_ value of 0.60 μM. Vero cells (2 × 10^3^ cells/well) were seeded with DMEM medium (2% FBS) into a 384-wellplate. After 24 h, 1000 fold serial dilution of the compound was added in triplicate to the 348-well plate. After incubation at 37 °C for 30 min, a twofold dilution 100× the 50% tissue culture infectious dose (TCID50) of EV71 virus in DMEM supplemented with 2% FBS was added to each well. The plate was incubated at 37 °C for 72–96 h when the viral control cells showed complete CPE, the cell survival was quantified using CCK-8. The A450 of the well was measured with a microtiter platereader (Envision, PerkinElmer, Waltham, MA, USA). The 50% inhibitory concentration (IC_50_) of the testing compound was calculated using the GraphPad Prism software.

## 4. Conclusions

The chemical investigation of the deep-sea-derived fungus *Penicillium* sp. SCSIO Ind16F01 has led to twelve compounds, including two new metabolites. Their structures were elucidated by the detailed analysis of spectroscopic data. All compounds were evaluated for their antiviral (H3N2 and EV71) and cytotoxic effects. Among them, compounds **2** and **8** exhibited anti-EV71 activities in vitro*,* with IC_50_ values of 81.6 and 19.8 μM. In addition, compound **8** also exhibited inhibitory activity against H3N2 with IC_50_ values of 24.1 μM, and cytotoxic effects on the tested cancer cell lines (K562, MCF-7, and SGC7901) with IC_50_ values of 16.6, 16.3, and 15.8 μM, respectively. Compound **8** was a unique cyclopentachromone dimer.

## Figures and Tables

**Table 1:** ^1^H (500 MHz) and ^13^C (125 MHz) NMR and HMBC data for **1** (DMSO-*d*_6_) and **2** (CDCl_3_).

Position	1		2	
	*δ*_H_ (*J* in Hz)	*δ*_C_ Type	*δ*_H_ (*J* in Hz)	*δ*_C_ Type
1		160.7 qC		161.4 qC
2	7.05 dd (0.7, 8.5)	110.0 CH	7.41 s	112.6 CH
3	7.72 t (8.5)	137.3 CH		135.7 qC
4	6.80 dd (0.7, 8.5)	107.2 CH	7.54 s	108.4 CH
4a		155.5 qC		156.9 qC
5		118.7 qC	3.20 m	30.2 CH_2_
			2.82 m	
5a		149.5 qC		175.1 qC
6		148.9 qC	2.32 m	27.6 CH_2_
			2.17 m	-
7		138.7 qC	4.94 d (6.5)	79.3 CH
7a				120.7 qC
8	7.57 d (1.0)	120.1 CH		181.0 qC
8a		115.2 qC		113.5 qC
9		180.5 qC		165.4 qC
9a		107.9 qC		
10	2.38 s	17.4 CH_3_	3.94 s	52.7 10-OMe
11		167.0 qC	3.49 s	57.5 11-OMe
12	3.87 s	52.3 11-OMe		
1-OH	12.32 s		12.64 s	

## Supplementary Materials

Supplementary materials are available online.

## References

[1] Kobayashi J.I. (2016). Search for new bioactive marine natural products and application to drug development. Chem. Pharm. Bull..

[2] El-Hossary E.M., Cheng C., Hamed M.M., Hamed A.N.E.-S., Ohlsen K., Hentschel U., Abdelmohsen U.R. (2017). Antifungal potential of marine natural products. Eur. J. Med. Chem..

[3] Schinke C., Martins T., Queiroz S.C.N., Melo I.S., Reyes F.G.R. (2017). Antibacterial compounds from marine bacteria, 2010–2015. J. Nat. Prod..

[4] Blunt J.W., Copp B.R., Keyzers R.A., Munro M.H.G., Prinsep M.R. (2017). Marine natural products. Nat. Prod. Rep..

[5] Skropeta D., Wei L. (2014). Recent advances in deep-sea natural products. Nat. Prod. Rep..

[6] Sumarah M.W., Puniani E., Blackwell B.A., Miller J.D. (2008). Characterization of polyketide metabolites from foliar endophytes of *Picea glauca*. J. Nat. Prod..

[7] Xia M.-W., Cui C.-B., Li C.-W., Wu C.-J., Peng J.-X., Li D.-H. (2015). Rare chromones from a fungal mutant of the marine-derived *Penicillium purpurogenum* G59. Mar. Drugs.

[8] Wang H.J., Gloer J.B., Scott J.A., Malloch D. (1995). Coniochaetones A and B New antifungal benzopyranones from the coprophilous fungus *Coniocheta saccardoi*. Tetrahedron Lett..

[9] Macias M., Gamboa A., Ulloa M., Toscano R.A., Mata R. (2001). Phytotoxic naphthopyranone derivatives from the coprophilous fungus *Guanomyces polythrix*. Phytochemistry.

[10] Trisuwan K., Rukachaisirikul V., Borwornwiriyapan K., Phongpaichit S., Sakayaroj J. (2014). Benzopyranone, benzophenone, and xanthone derivatives from the soil fungus *Penicillium citrinum* PSU-RSPG95. Tetrahedron Lett..

[11] Kuramata M., Fujioka S., Shimada A., Kawano T., Kimura Y. (2007). Citrinolactones A, B and C, and sclerotinin C, plant growth regulators from *Penicillium citrinum*. Biosci. Biotechnol. Biochem..

[12] Kong F.M., Carter G.T. (2003). Remisporine B, a novel dimeric chromenone derived from spontaneous Diels-Alder reaction of remisporine A. Tetrahedron Lett..

[13] Kakinuma N., Iwai H., Takahashi S., Hamano K., Yanagisawa T., Nagai K., Tanaka K., Suzuki K., Kirikae F., Kirikae T. (2000). Quinolactacins A, B and C: Novel quinolone compounds from *Penicillium* sp. EPF-6 I. Taxonomy, production, isolation and biological properties. J. Antibiot..

[14] Kim W.G., Song N.K., Yoo I.D. (2001). Quinolactacins A1 and A2, new acetylcholinesterase inhibitors from *Penicillium citrinum*. J. Antibiot..

[15] Clark B., Capon R.J., Lacey E., Tennant S., Gill J.H. (2006). Quinolactacins revisited: From lactams to imide and beyond. Org. Biomol. Chem..

[16] Takahashi S., Kakinuma N., Iwai H., Yanagisawa T., Nagai K., Suzuki K., Tokunaga T., Nakagawa A. (2000). Quinolactacins A, B and C: Novel quinolone compounds from *Penicillium* sp. EPF-6 II. Physico-chemical properties and structure elucidation. J. Antibiot..

[17] Bergeron R.J., Cavanaugh P.F., Kline S.J., Hughes R.G., Elliott G.T., Porter C.W. (1984). Antineoplastic and antiherpetic activity of spermidine catecholamide iron chelators. Biochem. Biophys. Res. Commun..

[18] Fang W., Lin X.P., Zhou X.F., Wan J.T., Lu X., Yang B., Ai W., Lin J., Zhang T.Y., Tu Z.C. (2014). Cytotoxic and antiviral nitrobenzoyl sesquiterpenoids from the marine-derived fungus *Aspergillus ochr**aceus* Jcma1F17. Medchemcomm.

[19] Ho H.Y., Cheng M.L., Weng S.F., Leu Y.L., Chiu D.T.Y. (2009). Antiviral effect of epigallocatechin gallate on enterovirus 71. J. Agric. Food Chem..

