# Supplementary material for: Xanthones and Quinolones Derivatives Produced by the Deep-Sea-Derived Fungus Penicillium sp. SCSIO Ind16F01
Source: Molecules. 2017 Dec 7;22(12):1999. doi: 10.3390/molecules22121999 (PMC6149711; doi:10.3390/molecules22121999)

Supporting Information

Xanthones and quinolones derivatives produced by the deep-sea-derived fungus *Penicillium* sp. SCSIO Ind16F01

Feng-an Liu [1,3], Xiuping Lin [2,3], Xuefeng Zhou [2], Minghao Chen [1], Xiuling Huang [1], Bin Yang [2,*] and Huaming Tao [1,*]

[1] School of Traditional Chinese Medicine, Southern Medical University, Guangzhou 510515, P. R. China; liufeng.an@outlook.com (F.L.); chen.minghao@haotmail.com (M.C.), 920205937@qq.com (X.H.)

[2] CAS Key Laboratory of Tropical Marine Bio-resources and Ecology / Guangdong Key Laboratory of Marine Materia Medica, South China Sea Institute of Oceanology, Chinese Academy of Sciences, Guangzhou 510301, P. R. China; xiupinglin@hotmail.com (X.L.); xfzhou@scsio.ac.cn (X.Z.)

[3] These two authors contributed equally to this work.

* Correspondence: taohm@smu.edu.cn (T. H.); bingo525@163.com (B.Y.); Tel.: +86-20-89023174

Academic Editor: name
Received: date; Accepted: date; Published: date

Abstract: Chemical investigation of the fungus *Penicillium* sp. SCSIO Ind16F01 derived from deep-sea sediment sample afforded a new xanthone, 3,8-dihydroxy -2-methyl-9-oxoxanthene-4-carboxylic acid methyl ester (**1**) and a new chromone, coniochaetone J (**2**), together with three known xanthones, 8-hydroxy-6-methyl-9-oxo-9H-xanthene-1-carboxylic acid methyl ester (**3**), 7,8-dihydroxy-6-methyl-9-oxo-9H-xanthene-1-carboxylic acid methyl ester (**4**), 1,6,8-trihydroxy-3-(hydroxymethyl)anthraquinone (**5**), three known chromones, coniochaetone B (**6**), citrinolactones B (**7**), epiremisporine B (**8**), and four reported rare class of *N*-methyl quinolone lactams: quinolactacins B (**9**), C1 (**10**), and C2 (**11**), and quinolonimide (**12**). The structures of new compounds were determined by analysis of the NMR and MS spectroscopic data. Those isolated compounds were evaluated for their antiviral (EV71 and H3N2) and cytotoxic activities.

Keywords: marine-derived fungus; *Penicillium* sp. SCSIO Ind16F01; xanthone; chromone; *N*-methyl quinolone lactams.

List of supporting information

Table S1. antiviral (H3N2 and EV71) and cytotoxic activities of Compounds **1**–**12**.

S1: ^{1}H-NMR (500 MHz, DMSO-*d*6) Spectrum of Compound **1**

S2: ^{13}C-NMR (125 MHz, DMSO-*d*6) Spectrum of Compound **1**

S3: Comparison the ^{1}H-NMR Spectrum of 1 in $CDCl_3$ and in DMSO-*d*6

S4: Comparison the ^{13}C-NMR Spectrum of 1 in $CDCl_3$ and in DMSO-*d*6

S5: HMQC (500 MHz) Spectrum of Compound **1**

S6: HMBC (500 MHz) Spectrum of Compound **1**

S7: HRESI-MS Spectrum of Compound **1**

S8: IR Spectrum of Compound **1**

S9: UV Spectrum of Compound **1**

S10: EI-MS Spectrum of Compound **1**

S11: ^{1}H-NMR (500 MHz, $CDCl_3$) Spectrum of Compound **2**

S12: ^{13}C-NMR (125 MHz, $CDCl_3$) Spectrum of Compound **2**

S13: HMQC (500 MHz) Spectrum of Compound **2**

S14: HMBC (500 MHz) Spectrum of Compound **2**

S15: HRESI-MS Spectrum of Compound **2**

S16: IR Spectrum of Compound **2**

S17: UV Spectrum of Compound **2**

S18: ^{1}H- and ^{13}C-NMR Spectrum of Compound **3**

S19: ^{1}H- and ^{13}C-NMR Spectrum of Compound **4**

S20: ^{1}H- and ^{13}C-NMR Spectrum of Compound **5**

S21: ^{1}H- and ^{13}C-NMR Spectrum of Compound **6**

S22: ^{1}H- and ^{13}C-NMR Spectrum of Compound **7**

S23: ^{1}H- and ^{13}C-NMR Spectrum of Compound **8**

S24: ^{1}H- and ^{13}C-NMR Spectrum of Compound **9**

S25: Comparing ^{1}H-NMR Spectrum of Compounds **9** and **10**

S26: ^{1}H- and ^{13}C-NMR Spectrum of Compound **11**

S27: ^{1}H- and ^{13}C-NMR Spectrum of Compound **12**

Table S1. antiviral (H3N2 and EV71) and cytotoxic activities of Compounds **1–12**

compounds	cytotoxic activities						anti-enterovirus 71		anti-H3N2	
	**K562**		**MCF-7**		**SGC7901**					
	Inhibition rate % (30μM)	IC50	Inhibition rate %(30μM)	IC50	Inhibition rate %(30μM)	IC50	Inhibition rate % (30μM)	IC50	Inhibition rate % (30μM)	IC50
1	97.88	ND	101.08	ND	85.29	ND	-12.21	ND	-4.9	ND
2	102.49	ND	96.48	ND	93.92	ND	37.25	8.16E-05	10.4	ND
3	107.53	ND	101.59	ND	103.80	ND	0.94	ND	-5.0	ND
4	104.51	ND	123.27	ND	107.97	ND	-5.93	ND	-6.0	ND
5	113.21	ND	109.80	ND	110.74	ND	8.50	ND	-0.9	ND
6	95.37	ND	104.47	ND	96.73	ND	5.59	ND	-4.1	ND
7	102.13	ND	121.25	ND	98.51	ND	6.68	ND	-3.3	ND
8	3.11	1.67E-05	-0.98	1.63E-05	3.46	1.58E-05	88.21	1.98E-05	91.5	2.41E-05
9	109.48	ND	104.61	ND	93.79	ND	6.76	ND	-0.7	ND
10	114.17	ND	120.96	ND	99.99	ND	11.49	ND	9.3	ND
11	111.19	ND	113.40	ND	104.15	ND	-2.74	ND	-5.3	ND
12	102.92	ND	105.77	ND	102.63	ND	1.13	ND	1.8	ND

S1: ^{1}H-NMR (500 MHz, DMSO-*d6*) Spectrum of Compound **1**

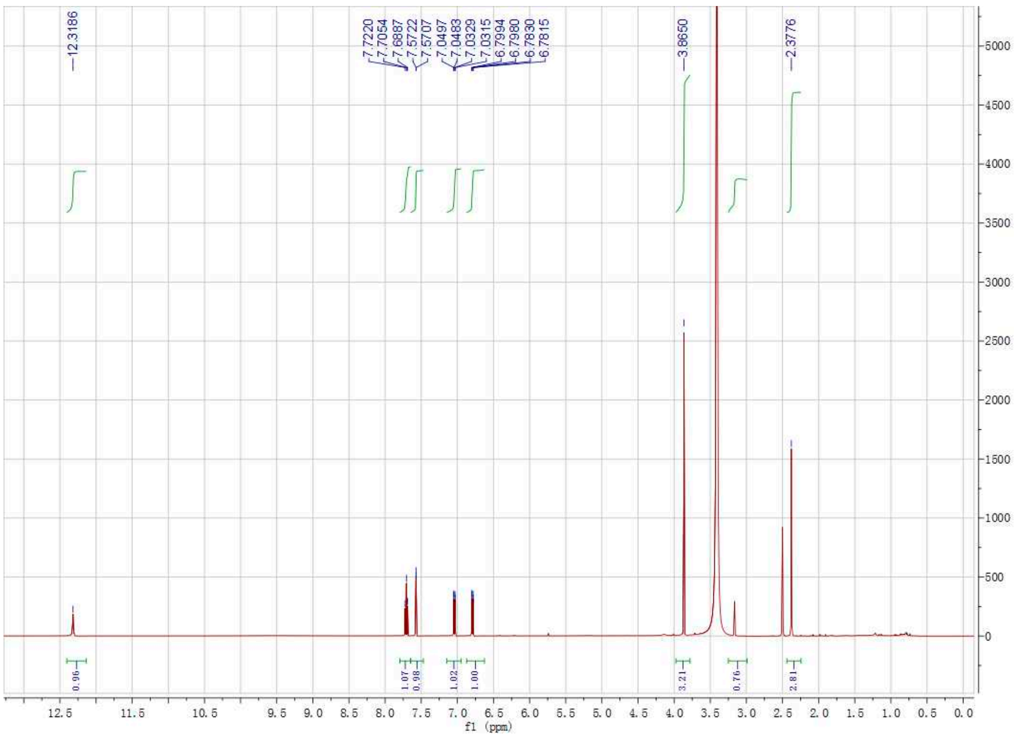

S2: ^{13}C-NMR (125 MHz, DMSO-*d6*) Spectrum of Compound **1**

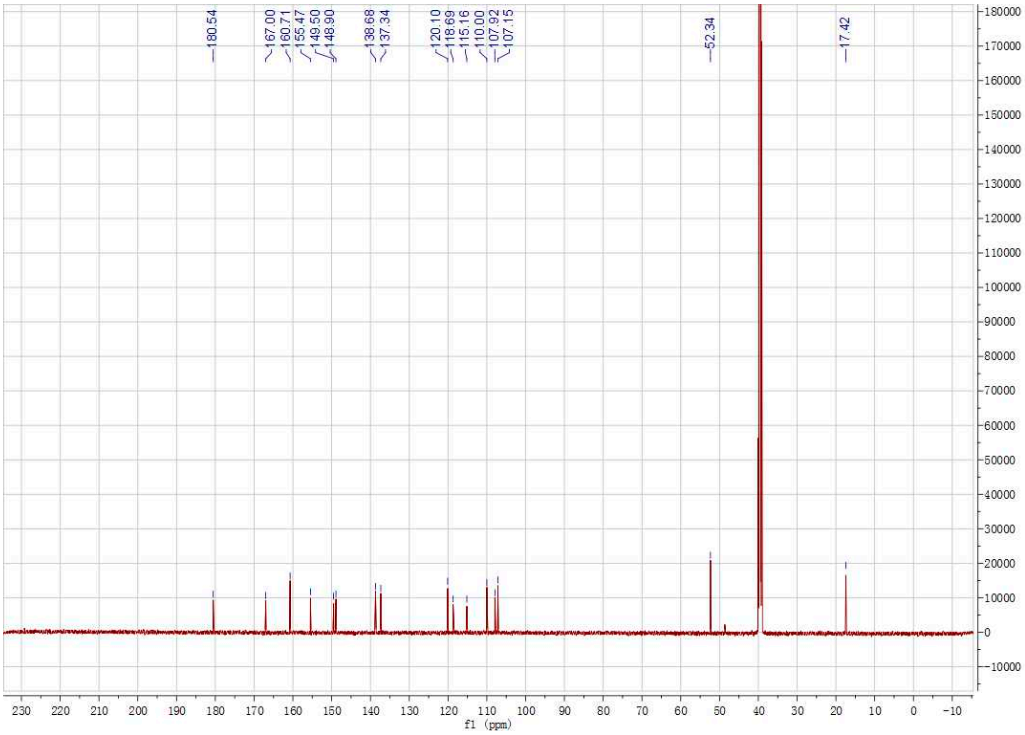

S3: Comparison the ^{1}H-NMR Spectrum of 1 in $CDCl_3$ and in DMSO-*d*6

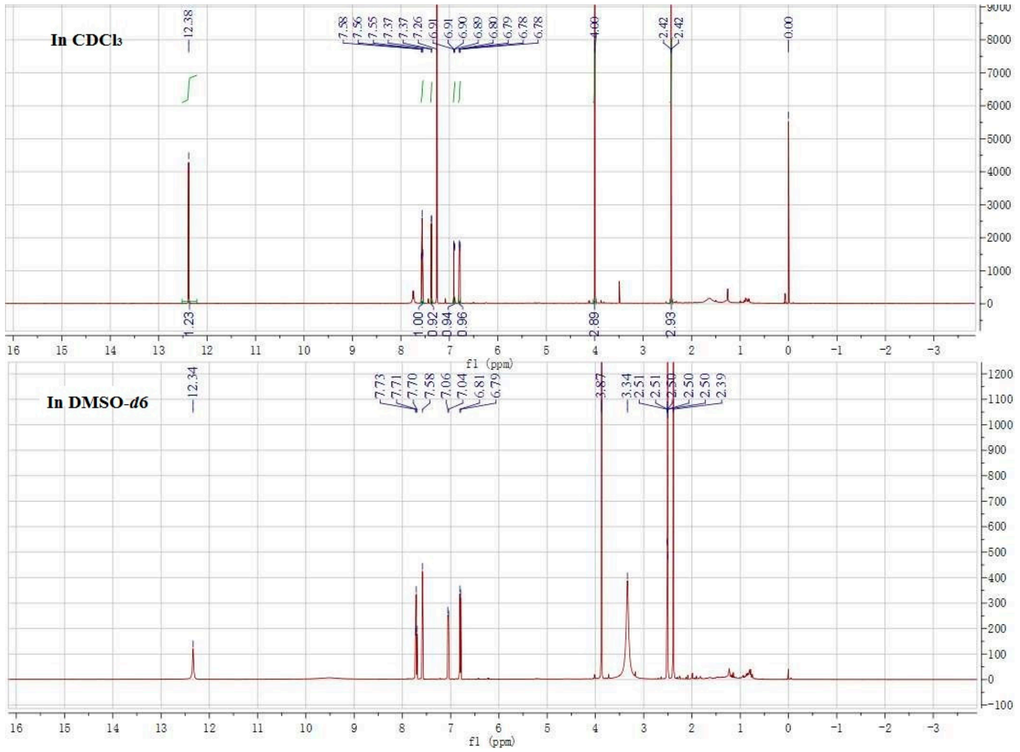

S4: Comparison the ^{13}C-NMR Spectrum of 1 in $CDCl_3$ and in DMSO-*d*6

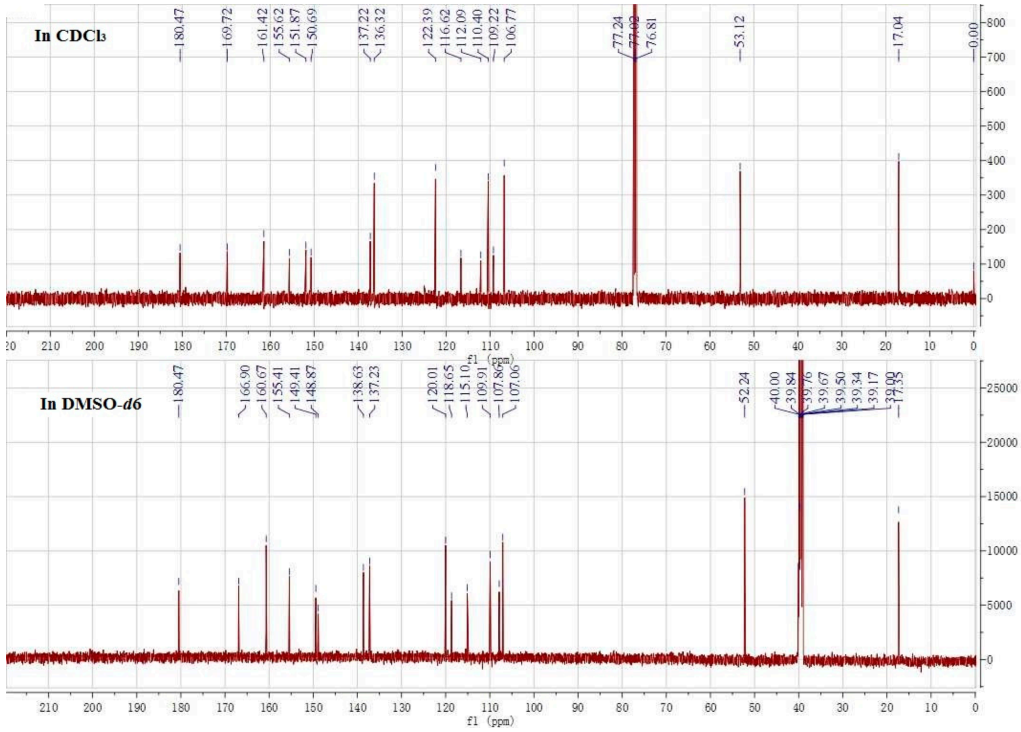

S5: HMQC (500 MHz) Spectrum of Compound **1**

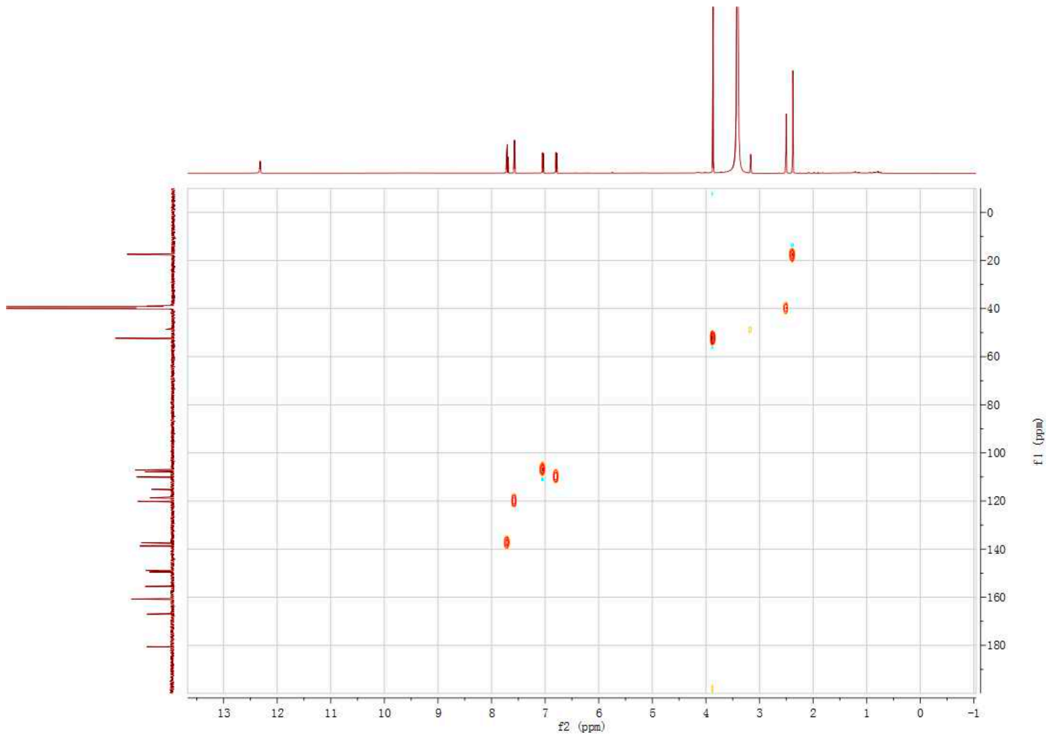

S6: HMBC (500 MHz) Spectrum of Compound **1**

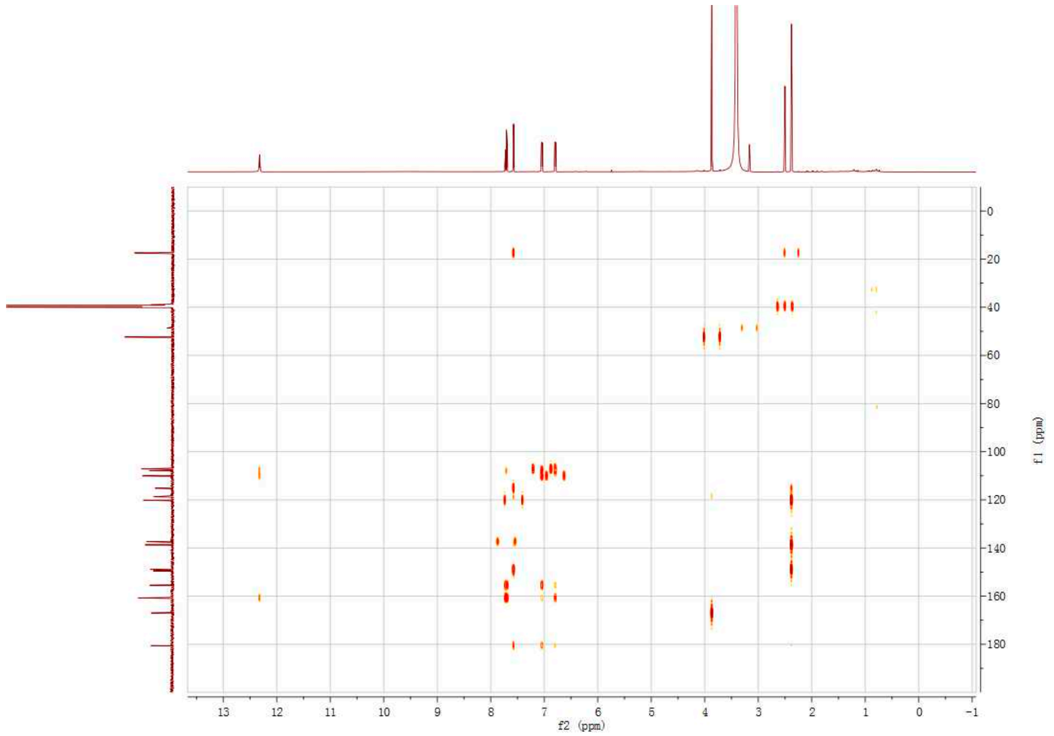

S7: HRESI-MS Spectrum of Compound **1**

Mass Spectrum SmartFormula Report

Analysis Info

Analysis Name	D:\Data\MS\data\201705\luoxiaowei_qmn-1_neg_3_01_3039.d	Acquisition Date	5/25/2017 9:43:27 AM
Method	LC_Direct Infusion_neg_100-1000mz.m	Operator	SCSIO
Sample Name	luoxiaowei_qmn-1_neg	Instrument	maXis 255552.00029
Comment			

Acquisition Parameter

Source Type	ESI	Ion Polarity	Negative	Set Nebulizer	0.4 Bar
Focus	Active	Set Capillary	4000 V	Set Dry Heater	180 °C
Scan Begin	100 m/z	Set End Plate Offset	-500 V	Set Dry Gas	4.0 l/min
Scan End	2000 m/z	Set Charging Voltage	0 V	Set Divert Valve	Waste
		Set Corona	0 nA	Set APCI Heater	0 °C

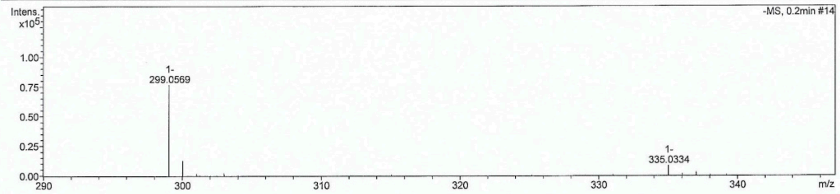

Meas. m/z	#	Ion Formula	Score	m/z	err [ppm]	err [mDa]	mSigma	rdb	e⁻ Conf	N-Rule
299.056853	1	C16H11O6	100.00	299.056112	-2.5	-0.7	4.9	11.5	even	ok
335.033385	1	C16H12ClO6	100.00	335.032789	-1.8	-0.6	13.5	10.5	even	ok
635.096323	1	C32H24ClO12	100.00	635.096177	0.2	0.1	94.3	20.5	even	ok

luoxiaowei_qmn-1_neg_3_01_3039.d

Bruker Compass DataAnalysis 4.1 printed: 5/25/2017 9:59:27 AM by: SCSIO Page 1 of 1

S8: IR Spectrum of Compound **1**

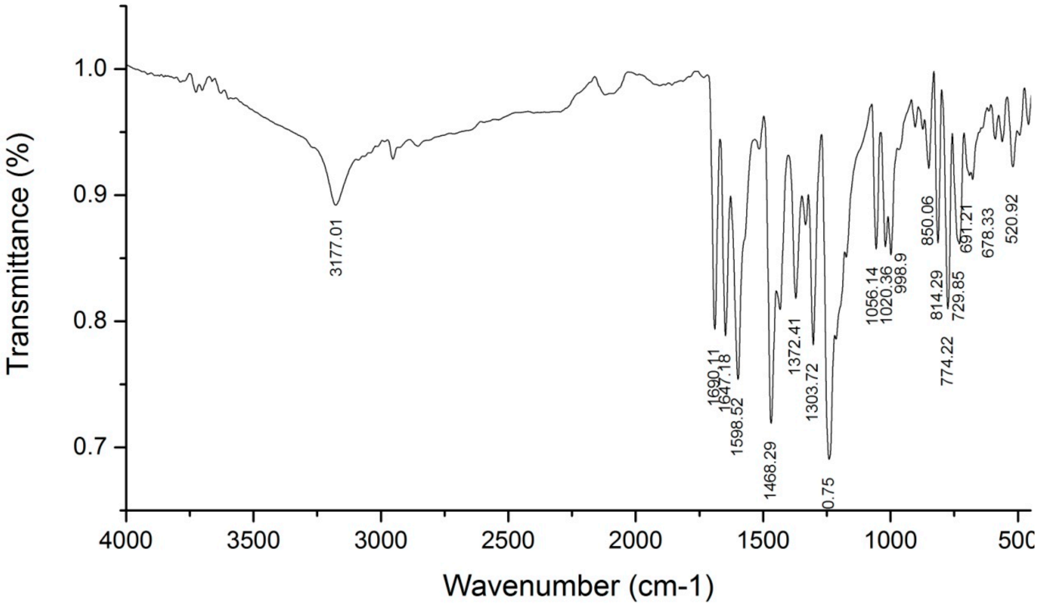

S9: UV Spectrum of Compound **1**

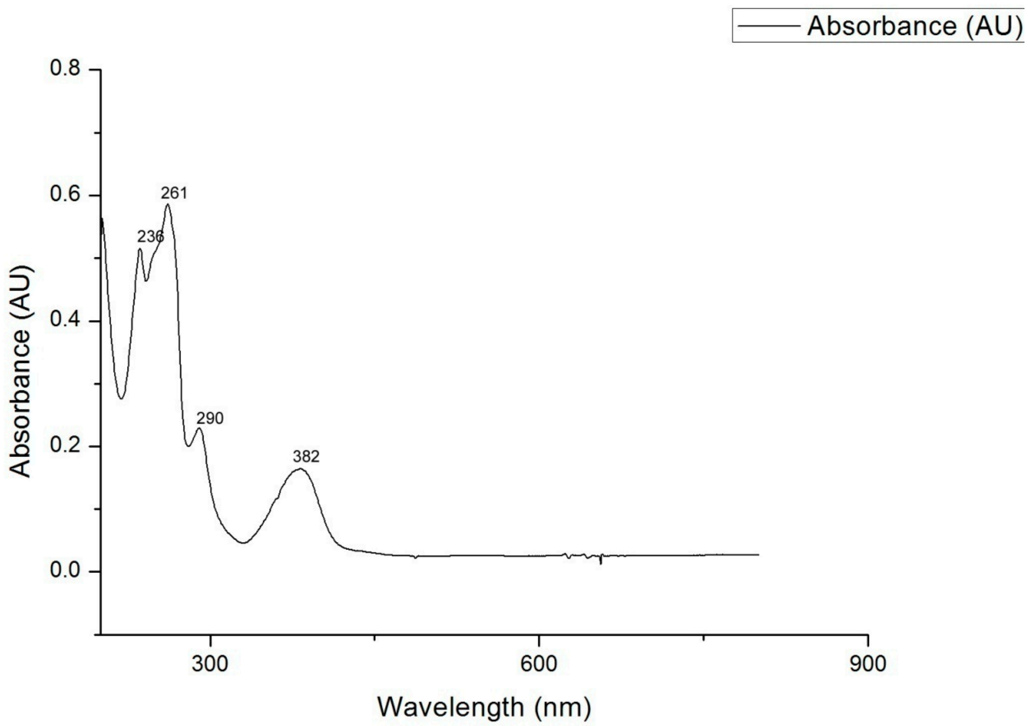

S10: EI-MS Spectrum of Compound **1**

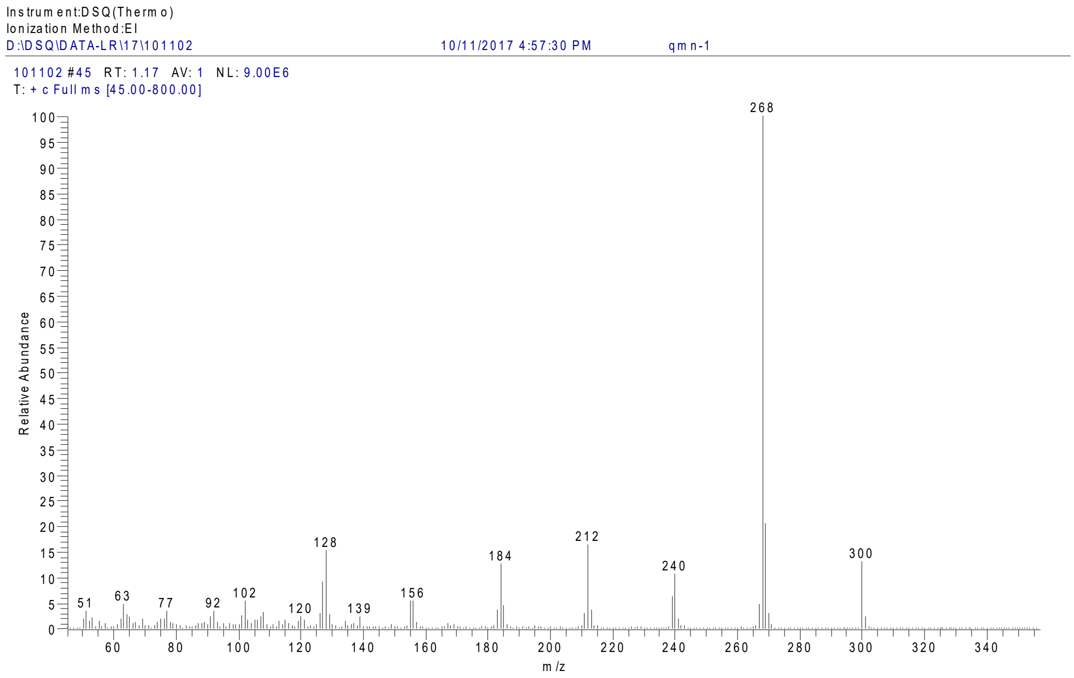

S11: ^{1}H-NMR (500 MHz, $CDCl_3$) Spectrum of Compound **2**

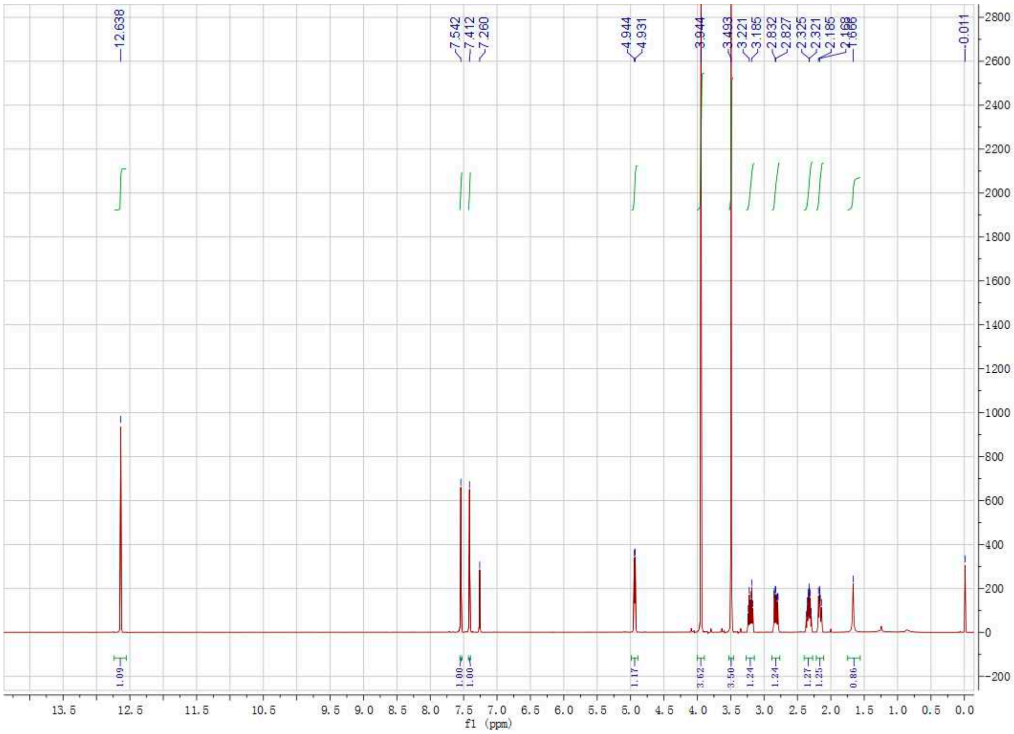

S12: ^{13}C-NMR (125 MHz, $CDCl_3$) Spectrum of Compound **2**

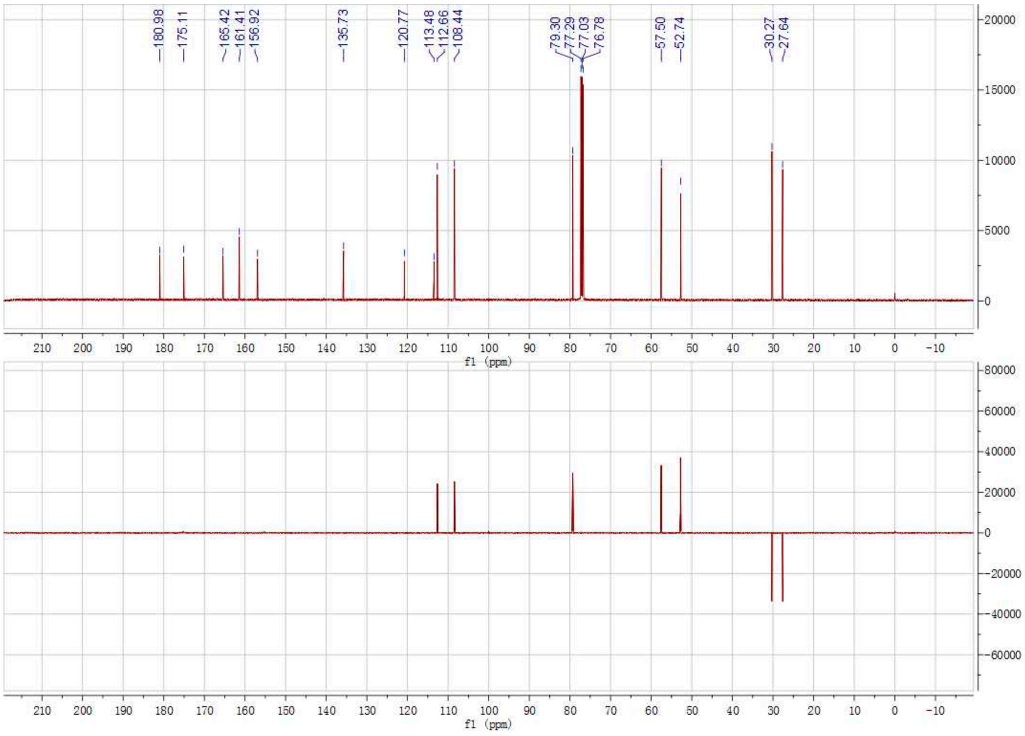

S13: HMQC (500 MHz) Spectrum of Compound **2**

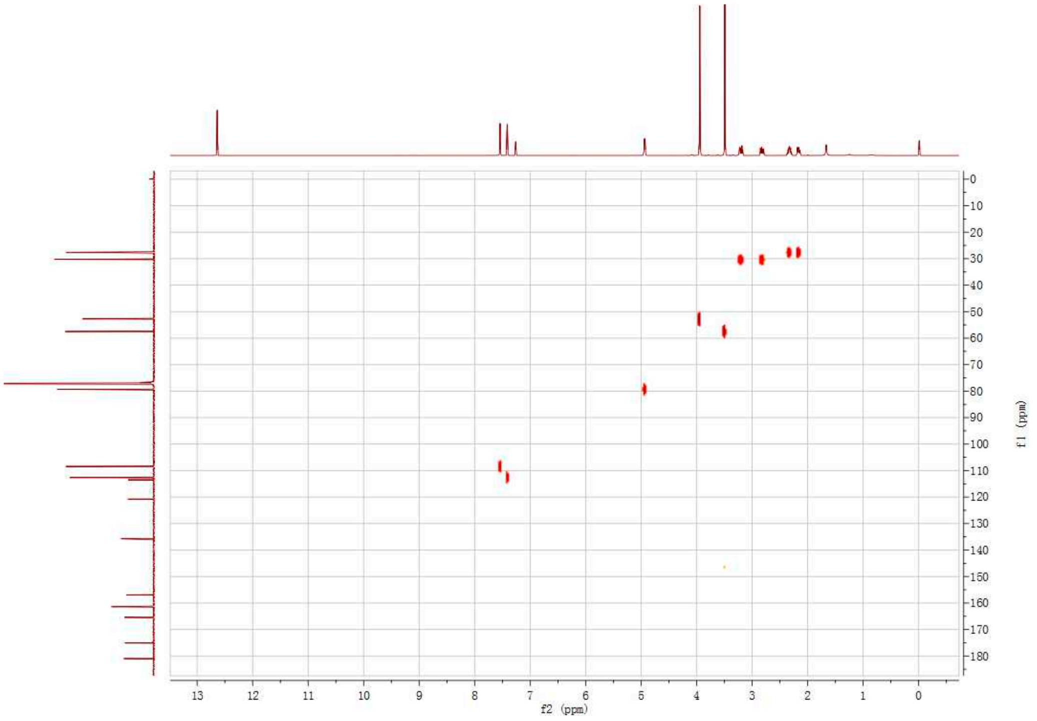

S14: HMBC (500 MHz) Spectrum of Compound **2**

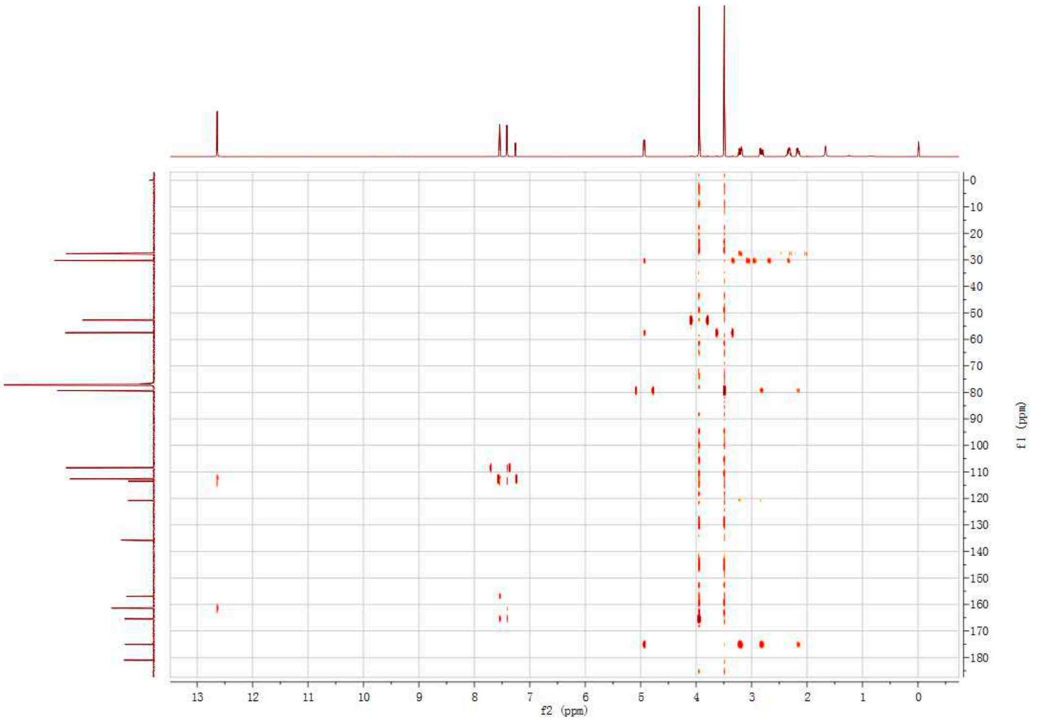

S15: HRESI-MS Spectrum of Compound **2**

Mass Spectrum SmartFormula Report

Analysis Info

Analysis Name	D:\Data\MS\data\201606\yangbin_IFB-1_pos_2_01_1947.d	Acquisition Date	6/3/2016 11:13:30 AM
Method	LC_Direct Infusion_pos_100-1000mz.m	Operator	SCSIO
Sample Name	yangbin_IFB-1_pos	Instrument / Ser#	maXis 29
Comment			

Acquisition Parameter

Source Type	ESI	Ion Polarity	Positive	Set Nebulizer	0.4 Bar
Focus	Active	Set Capillary	4500 V	Set Dry Heater	180 °C
Scan Begin	100 m/z	Set End Plate Offset	-500 V	Set Dry Gas	4.0 l/min
Scan End	2000 m/z	Set Collision Cell RF	800.0 Vpp	Set Divert Valve	Waste

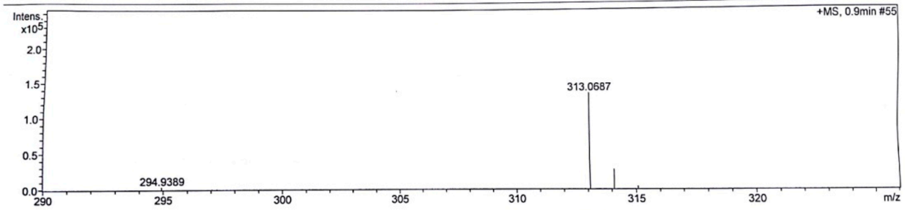

Meas. m/z	#	Formula	Score	m/z	err [ppm]	err [mDa]	mSigma	rdb	e⁻ Conf	N-Rule
313.0687	1	C 15 H 14 Na O 6	97.99	313.0683	-1.3	-0.4	23.8	8.5	even	ok
603.1473	1	C 30 H 28 Na O 12	100.00	603.1473	-0.0	-0.0	7.4	16.5	even	ok

Bruker Compass DataAnalysis 4.0 printed: 6/3/2016 11:19:55 AM Page 1 of 1

S16: IR Spectrum of Compound **2**

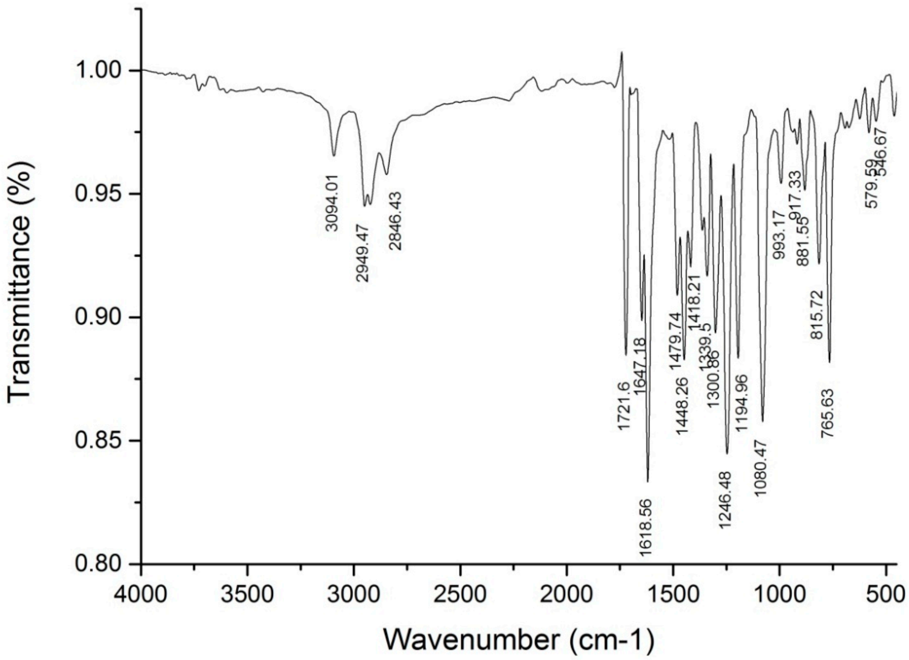

S17: UV Spectrum of Compound **2**

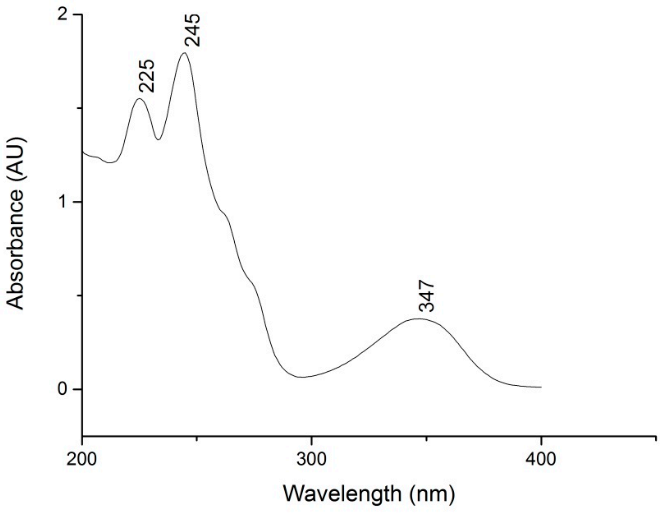

S18: ^{1}H- and ^{13}C-NMR Spectrum of Compound **3**

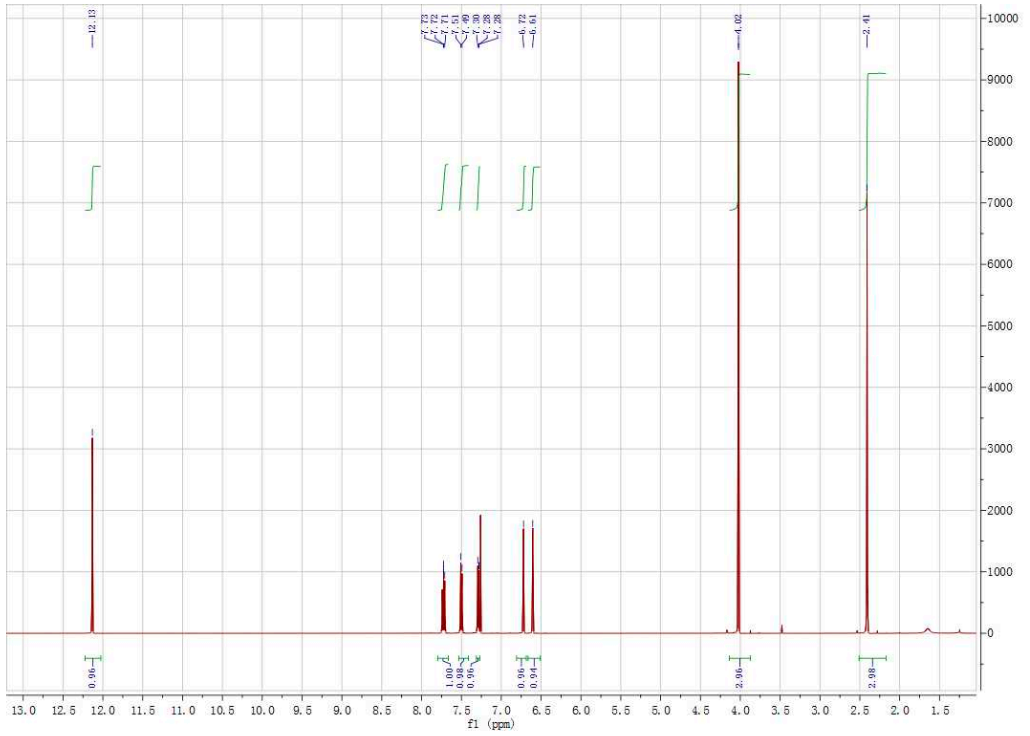

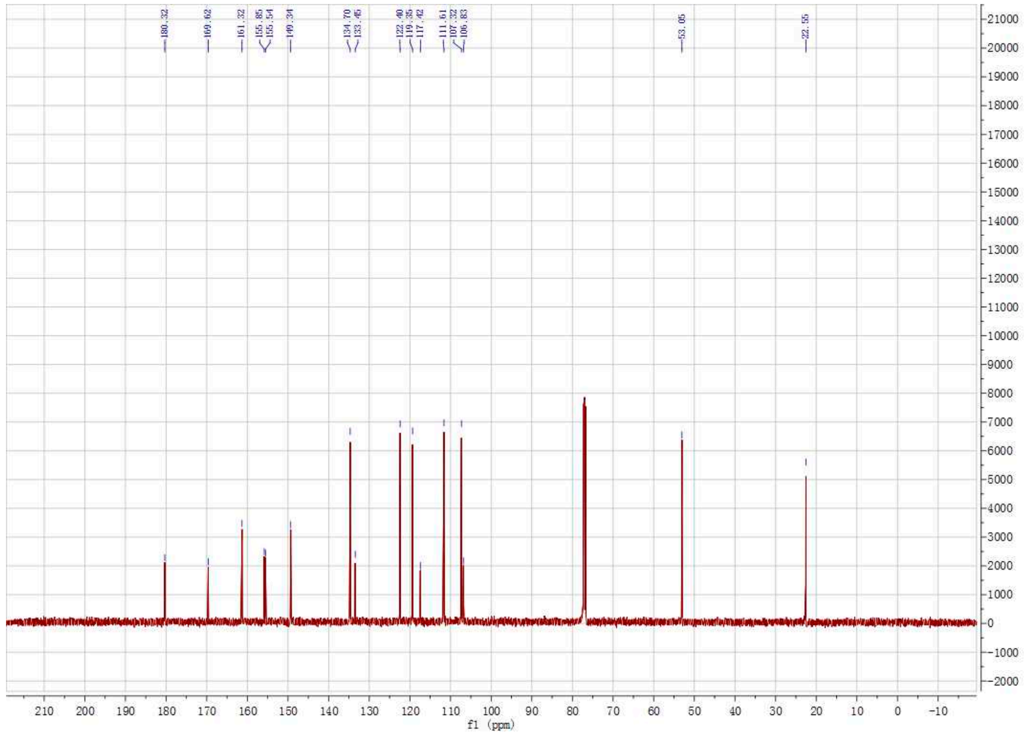

S19: ^{1}H- and ^{13}C-NMR Spectrum of Compound **4**

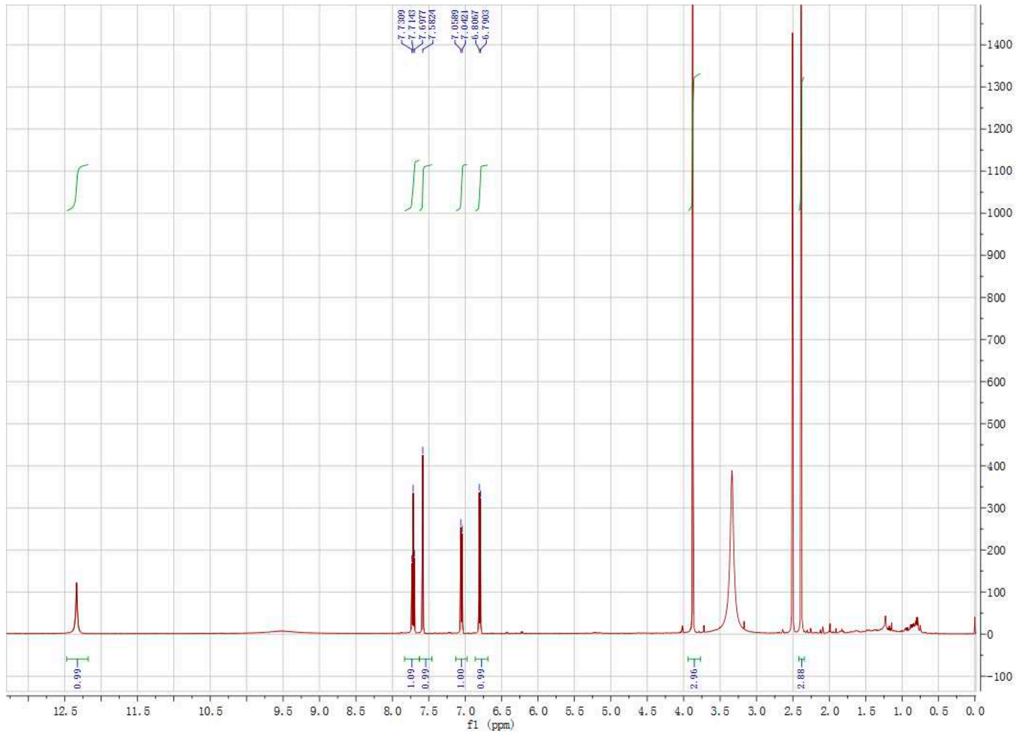

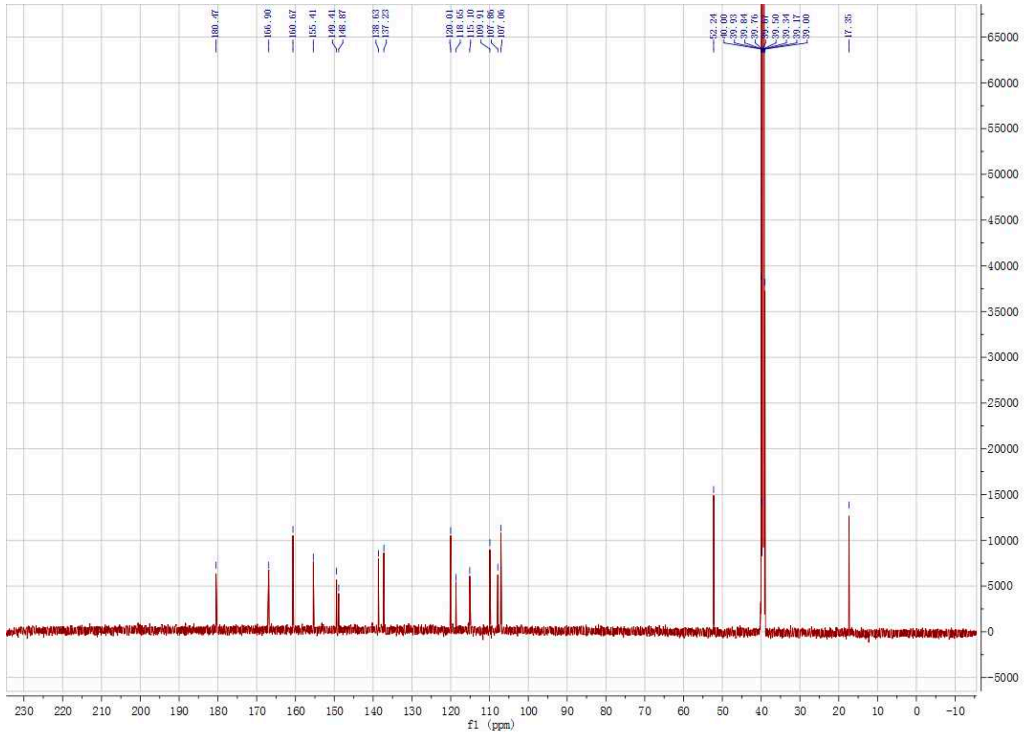

S20: ^{1}H- and ^{13}C-NMR Spectrum of Compound **5**

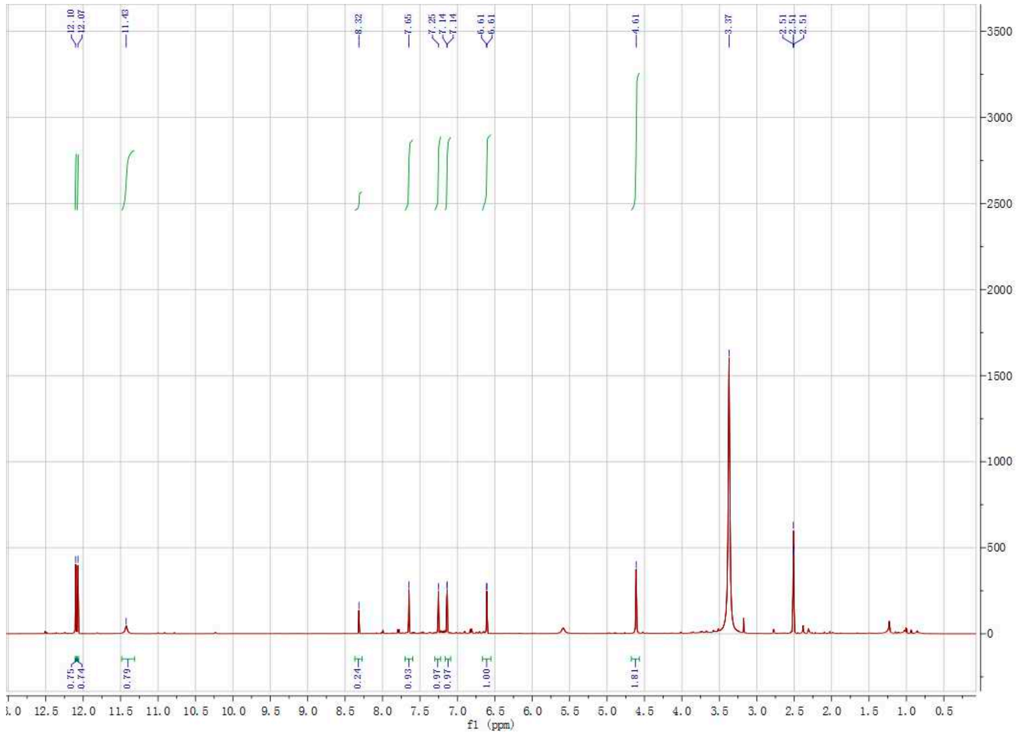

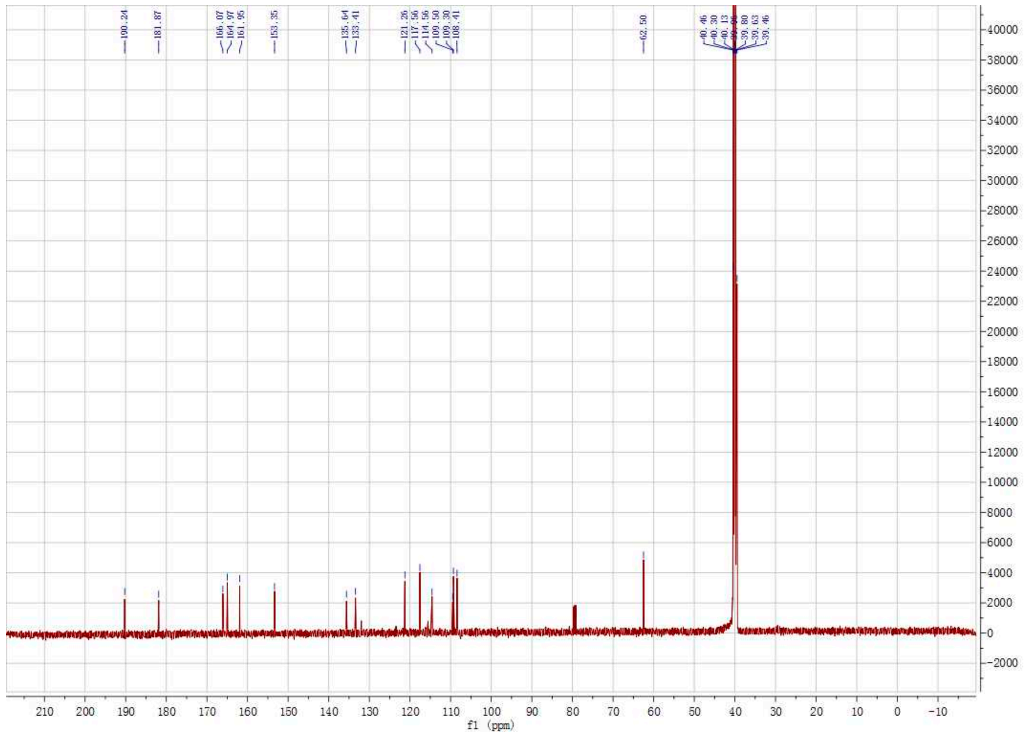

S21: ^{1}H- and ^{13}C-NMR Spectrum of Compound **6**

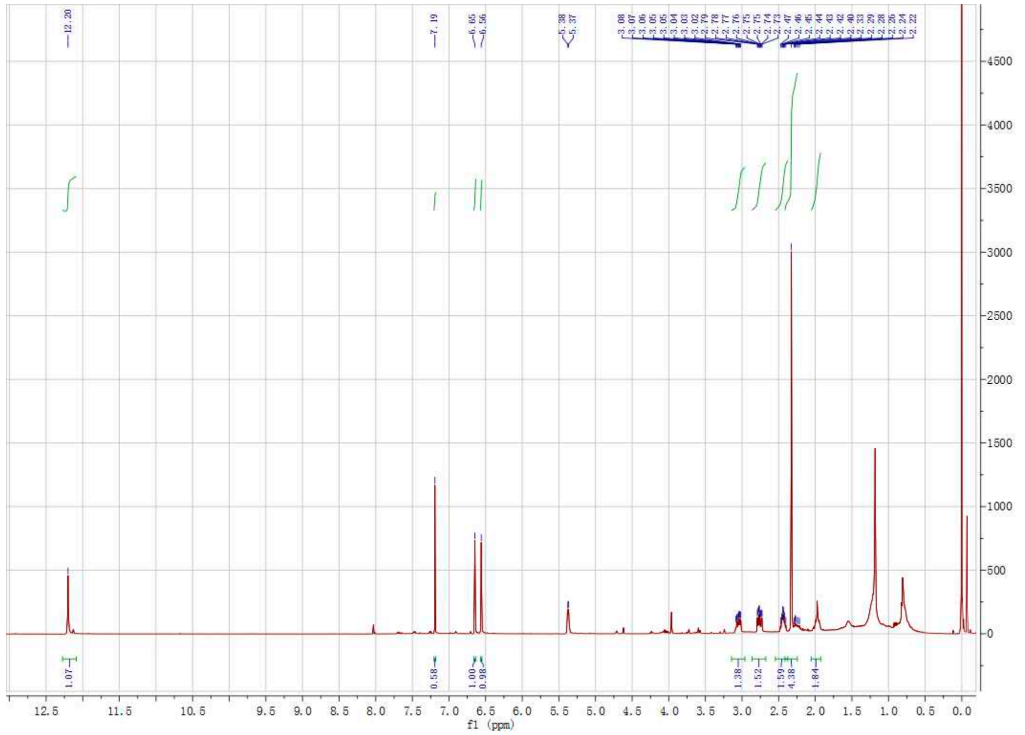

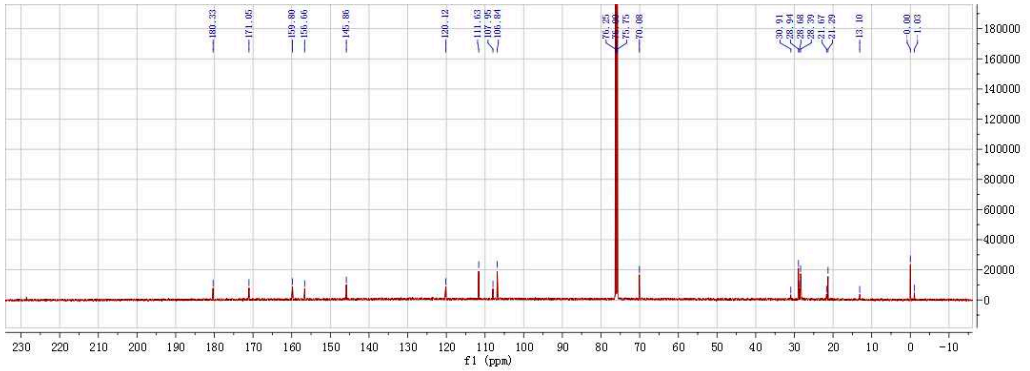

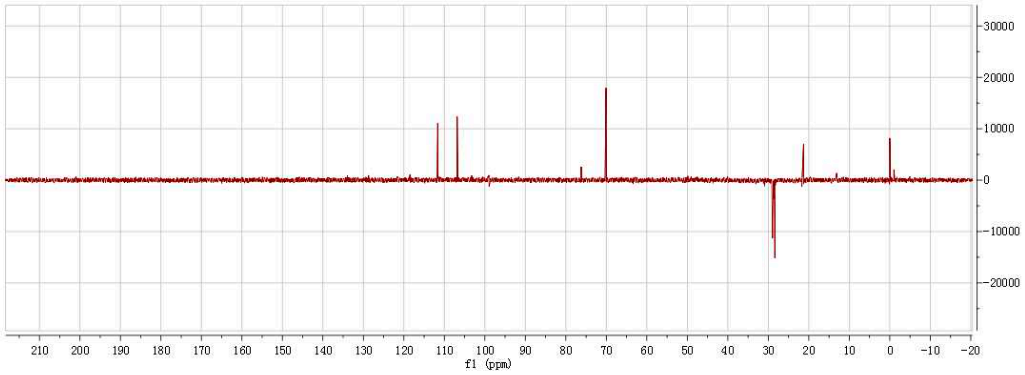

S22: ^{1}H- and ^{13}C-NMR Spectrum of Compound **7**

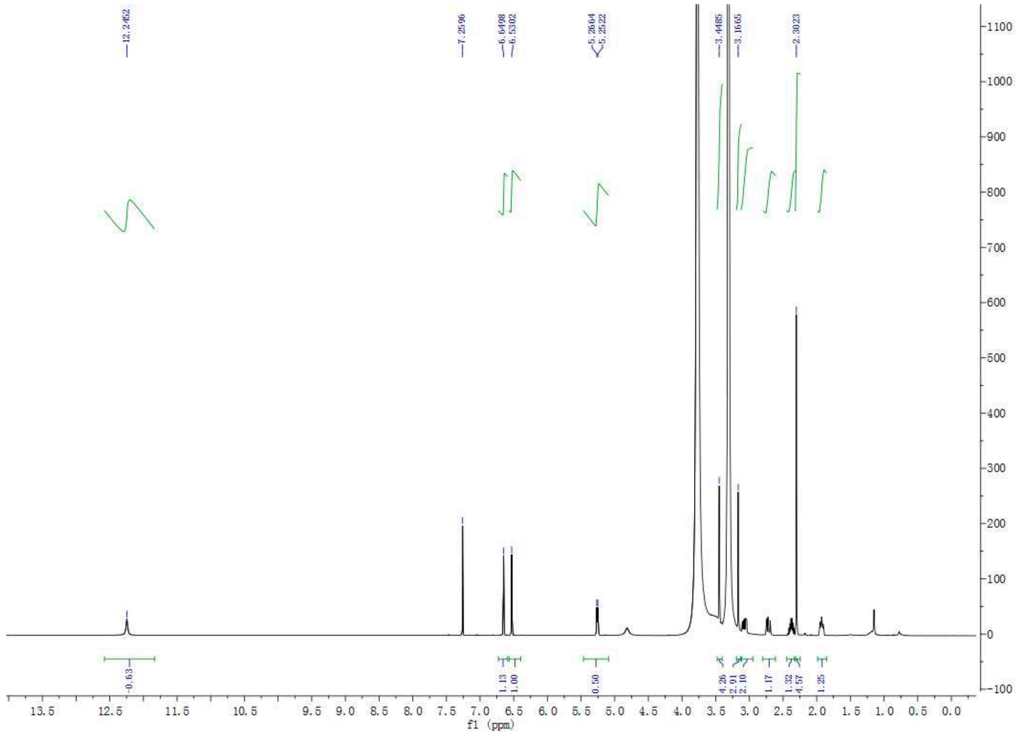

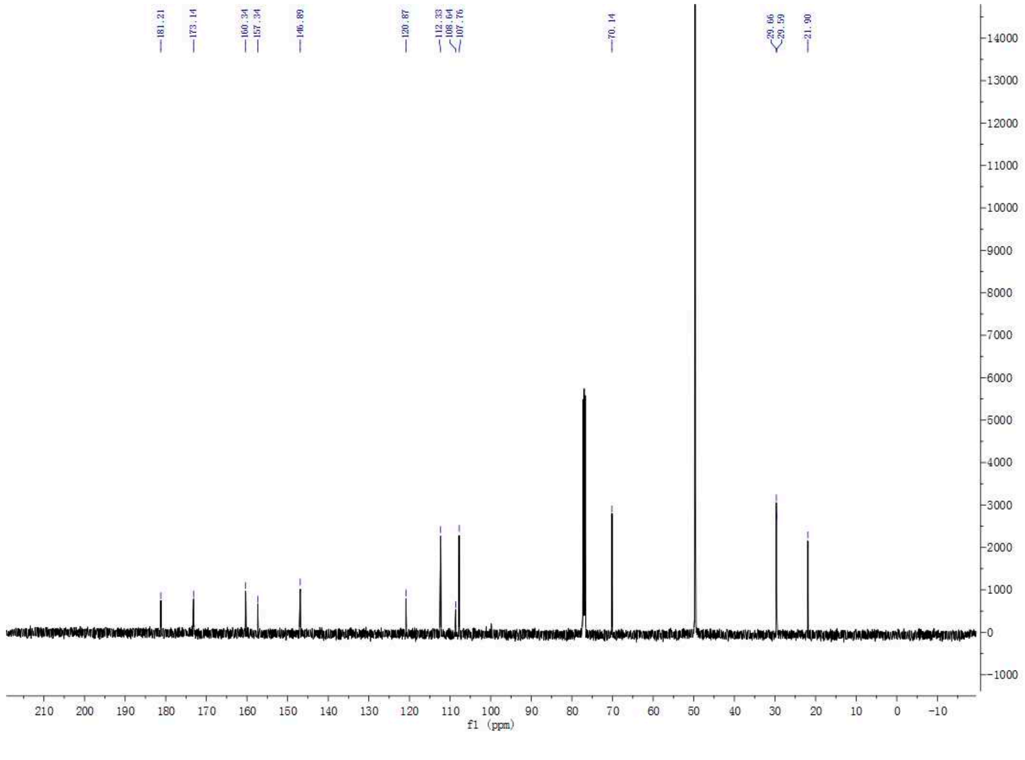

S23: ^{1}H- and ^{13}C-NMR Spectrum of Compound **8**

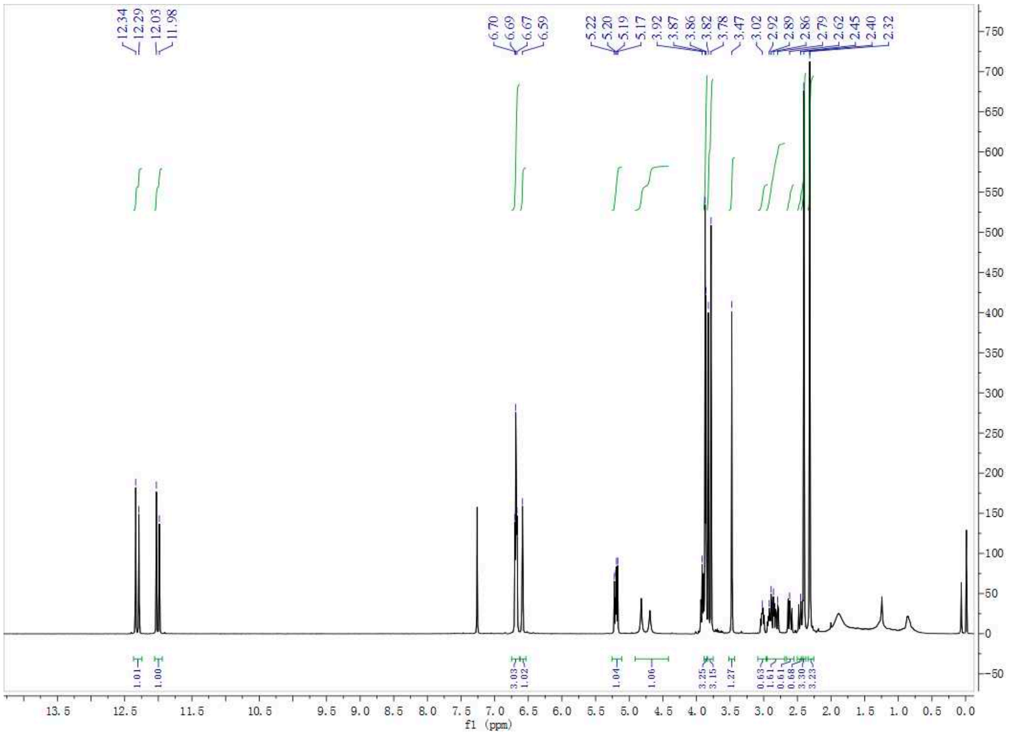

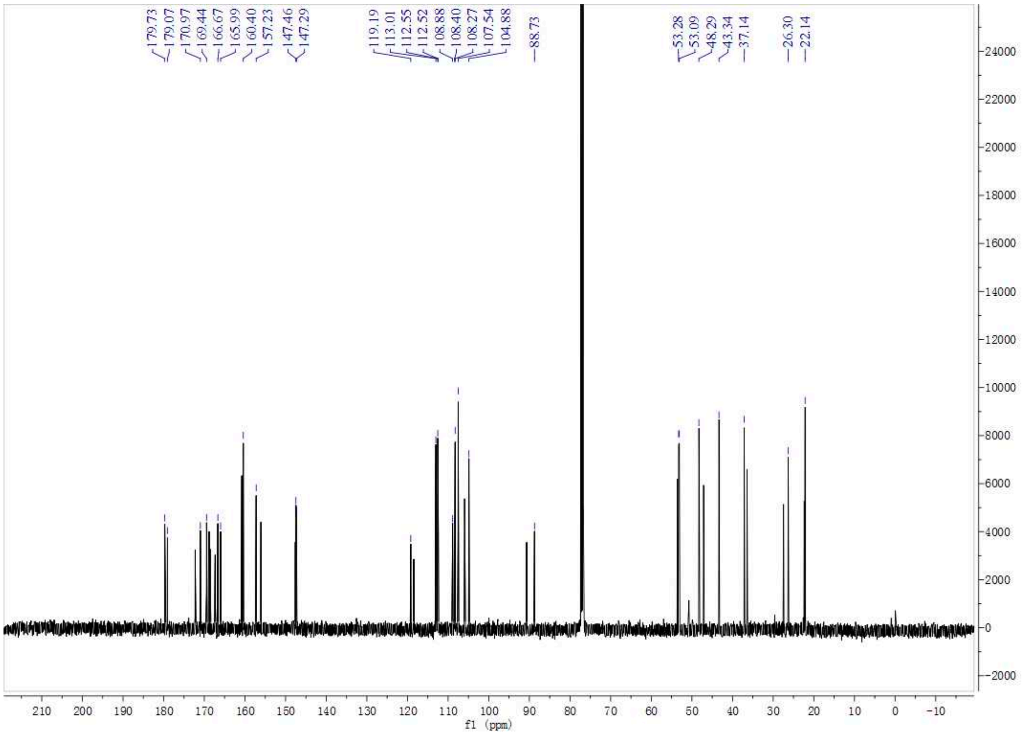

S24: ^{1}H- and ^{13}C-NMR Spectrum of Compound **9**

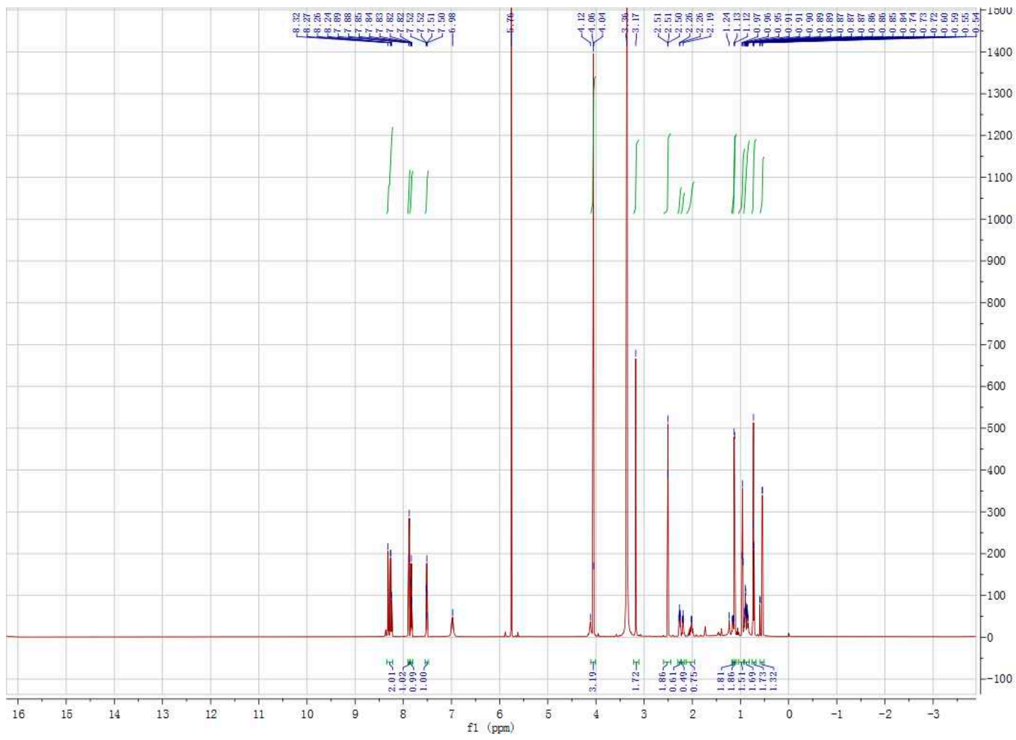

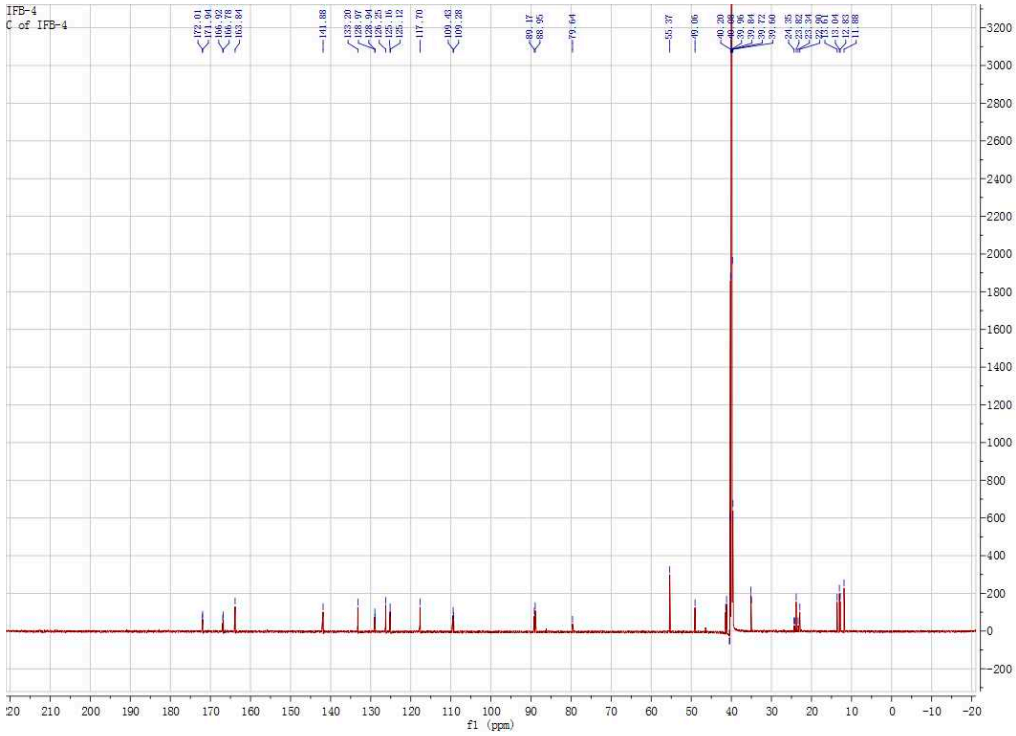

S25: Comparing ^{1}H-NMR Spectrum of Compounds **9** and **10**

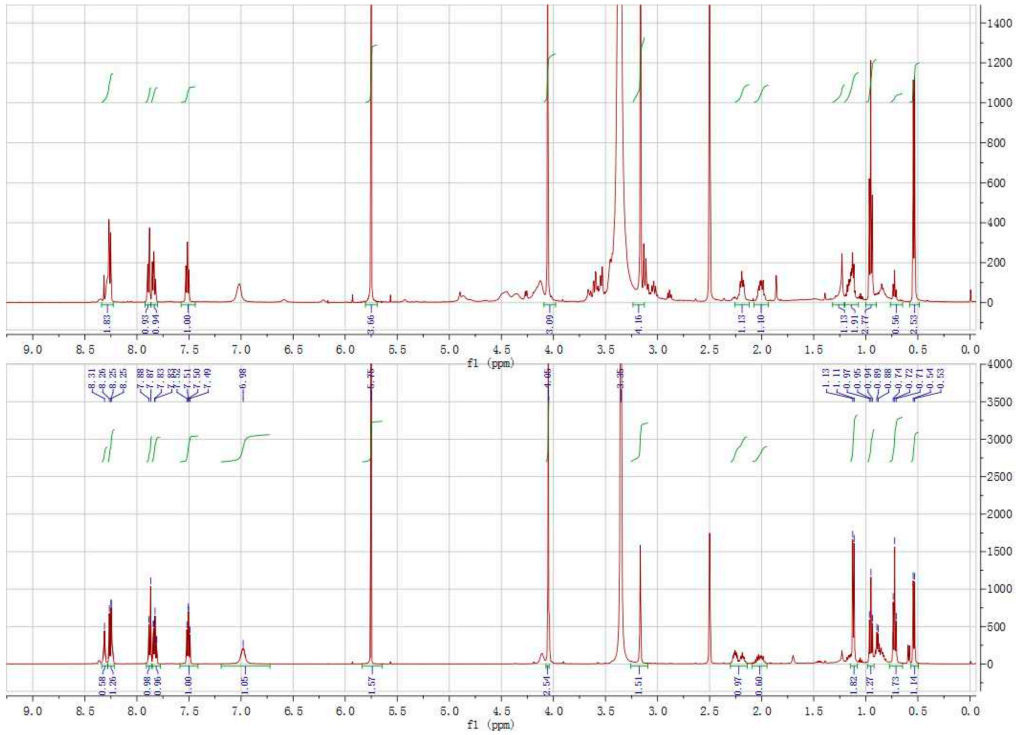

S26: ^{1}H- and ^{13}C-NMR Spectrum of Compound **11**

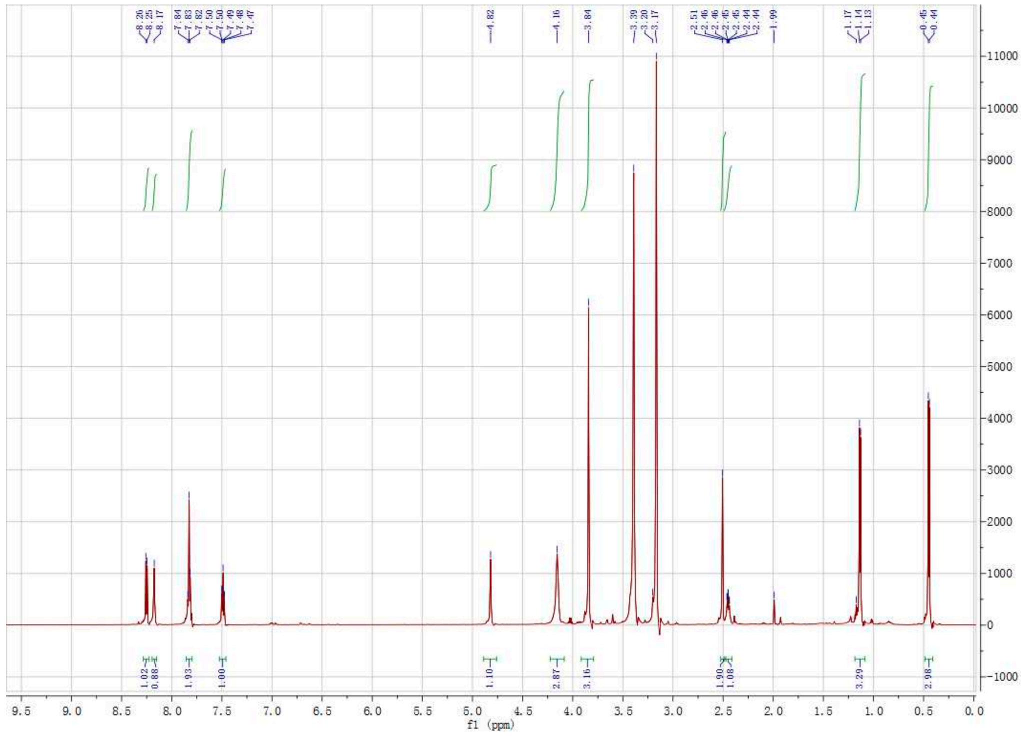

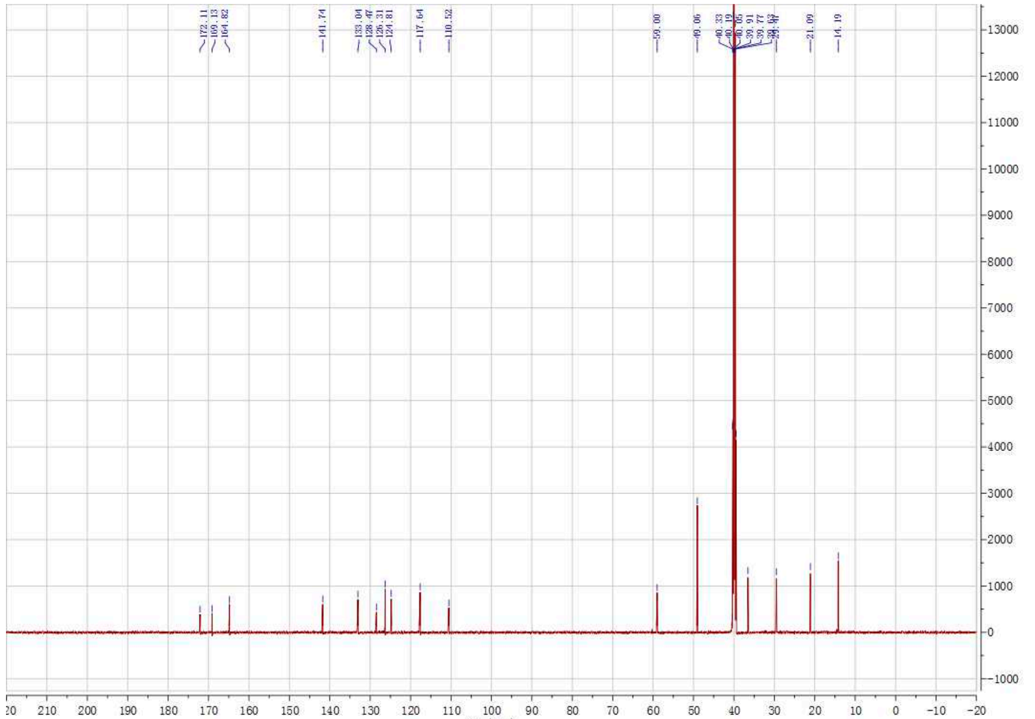

S27: ^{1}H- and ^{13}C-NMR Spectrum of Compound **12**

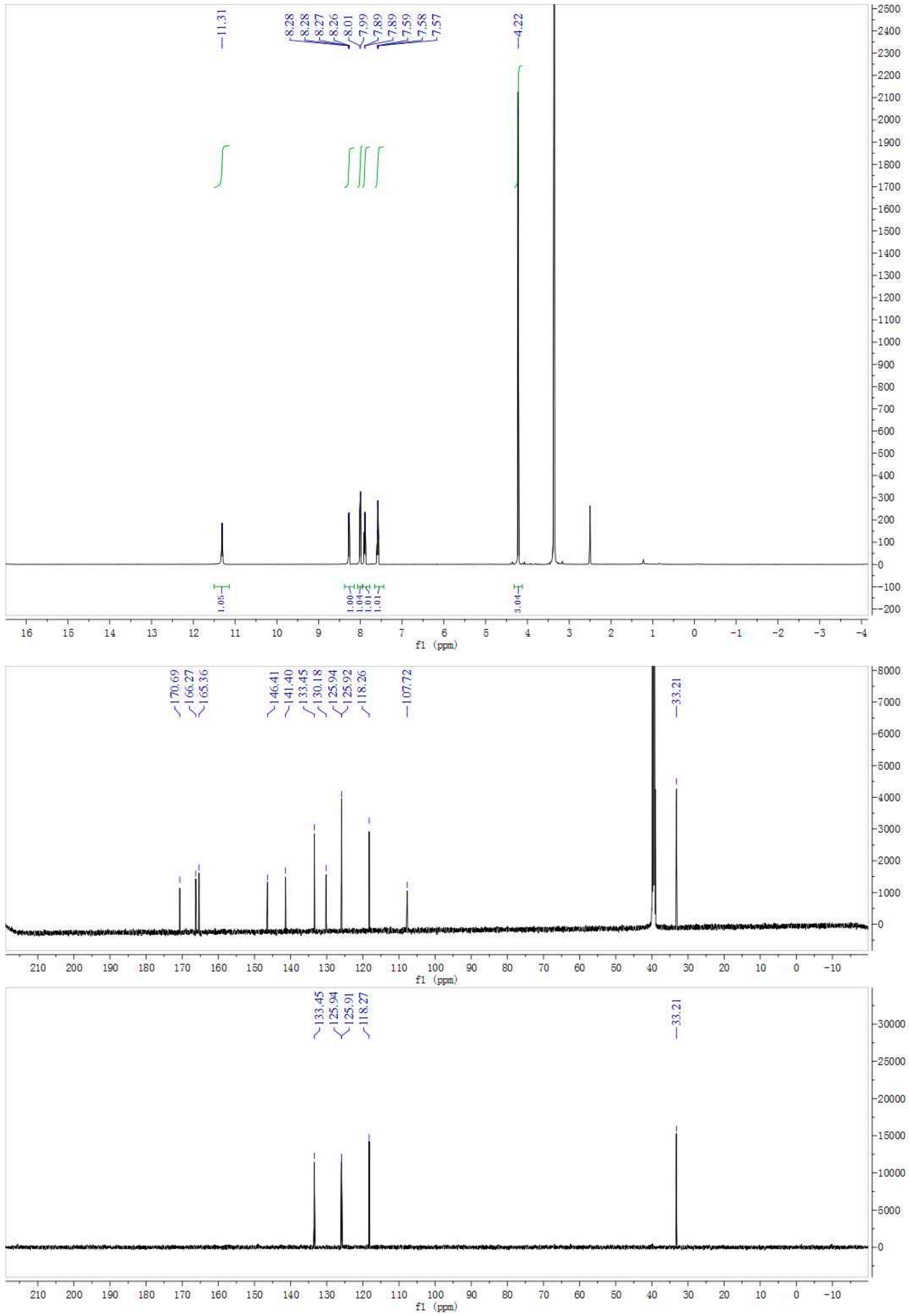

Supplement: Supplementary file 1 [file molecules-22-01999-s001.pdf]
